# CD95/Fas ligand mRNA is toxic to cells through more than one mechanism

**DOI:** 10.1186/s43556-023-00119-1

**Published:** 2023-04-15

**Authors:** Ashley Haluck-Kangas, Madelaine Fink, Elizabeth T. Bartom, Marcus E. Peter

**Affiliations:** 1 Department of Medicine/Division Hematology/Oncology, Feinberg School of Medicine, Chicago, IL USA; 2 Department of Biochemistry and Molecular Genetics, Chicago, IL USA; 3 Department of Preventive Medicine/Division of Biostatistics, Feinberg School of Medicine, Northwestern University, Chicago, IL USA

**Keywords:** RNAi, FasL, RISC, Cell death, RNA toxicity

## Abstract

CD95/Fas ligand (CD95L) induces apoptosis through protein binding to the CD95 receptor. However, CD95L mRNA also induces toxicity in the absence of CD95 through induction of DISE (Death Induced by Survival Gene Elimination), a form of cell death mediated by RNA interference (RNAi). We now report that CD95L mRNA processing generates a short (s)RNA nearly identical to shL3, a commercial CD95L-targeting shRNA that led to the discovery of DISE. Neither of the miRNA biogenesis proteins Drosha nor Dicer are required for this processing. Interestingly, CD95L toxicity depends on the core component of the RISC, Ago2, in some cell lines, but not in others. In the HCT116 colon cancer cell line, Ago 1–4 appear to function redundantly in RNAi. In fact, Ago 1/2/3 knockout cells retain sensitivity to CD95L mRNA toxicity. Toxicity was only blocked by mutation of all in-frame start codons in the CD95L ORF. Dying cells exhibited an enrichment of RISC bound (R)-sRNAs with toxic 6mer seed sequences, while expression of the non-toxic CD95L mutant enriched for loading of R-sRNAs with nontoxic 6mer seeds. However, CD95L is not the only source of these R-sRNAs. We find that CD95L mRNA may induce DISE directly and indirectly, and that alternate mechanisms may underlie CD95L mRNA processing and toxicity.

**Supplementary Information:**

The online version contains supplementary material available at 10.1186/s43556-023-00119-1.

## Introduction

CD95/Fas ligand (CD95L) is a well-established inducer of extrinsic apoptosis [1–3]. It is expressed by activated T cells and maintains T cell homeostasis by negatively regulating clonal expansion. In cancer, CD95L expression is induced upon exposure to genotoxic agents, and has been implicated in death by chemotherapeutics and radiation therapy [4–7]. When membrane bound CD95L binds to its cognate receptor, CD95, it promotes trimerization of CD95 inducing the recruitment of proteins that, in sensitive cells, activate caspases that execute apoptosis [8, 9]. However, we recently reported that overexpression of CD95L induces cell death even in the absence of CD95 receptor [10]. Disrupting engagement of CD95 signaling both by CRISPR/Cas9 knock-out of CD95 or by mutation of CD95L did not block this form of cell death. Cell death occurred in a caspase independent fashion, and exhibited features of Death Induced by Survival gene Elimination (DISE) [11].

DISE results from the RNA interference (RNAi)-mediated downregulation of networks of genes required for cell survival. Targeting of these essential survival genes depends upon the 6mer seed sequence, nucleotides 2–7, of a short RNA (sRNA) in the RNA-Induced Signaling Complex (RISC). DISE is characterized by the generation of ROS, and the accumulation of DNA damage. Dying cells exhibit morphological and biochemical features of apoptosis, autophagy, necroptosis, and mitotic catastrophe [12]. It was discovered through experiments using commercial si-/shRNAs designed to target CD95 and CD95L. About 80% of these sequences induced death even when the target site was deleted [11]. Through a series of experiments, we determined that these si-/shRNAs engaged in miRNA-like seed-based targeting of hundreds of survival genes. Based on the observation that only six nucleotides of the seed sequence (positions 2–7) were required to mediate this targeting [11], we performed arrayed screens of all 4,096 possible permutations of six nucleotide seed sequences incorporated into a non-targeting siRNA backbone expressed in three human and three murine cell lines [10, 13] (6merdb.org). The effect these sequences exerted on viability was averaged between cell lines by species and is referred to as the seed viability.

Based on the data from these seed viability screens, predictions can now be made regarding the effect an sRNA will have on cell viability through 6mer seed-based targeting mediated by the RISC. We developed a gene agnostic small RNA-seq analysis pipeline, called SPOROS, to make these predictions [14]. The pipeline analyzes small RNA-Seq data by tallying reads with the same 6mer seed sequence and attaching the associated seed viability data. Both the overall 6mer seed viability of all sequences and differentially expressed sequences can then be determined. We found that small shifts in the 6mer seed viability of Ago bound sRNAs can predict cellular responses to toxic stimuli [15].

We previously reported that CD95L mRNA induces toxicity at least in part through RNAi [16]. In addition to inducing morphological features of DISE, expression of CD95L promoted similar gene expression changes observed in DISE. Sequencing of RISC bound sRNAs (R-sRNAs) revealed that CD95L mRNA was processed and CD95L-derived sRNAs were loaded into the RISC. A similar processing and RISC loading of endogenous mRNAs was also observed with many endogenous mRNAs related to protein translation enriched in the RISC. All these R-sRNAs were more abundant in cells lacking the miRNA biogenesis enzyme Drosha, an observation we interpreted as resulting from a global downregulation of miRNAs and thus reduced competition for RISC loading. Interestingly, Drosha k.o. cells and cells lacking another critical miRNA processing enzyme, Dicer, were also more sensitive to CD95L toxicity, supporting our hypothesis that miRNAs may protect the RISC from the loading of toxic sequences. Knockdown of Ago2, the primary mediator of RNAi, rescued the toxicity in an ovarian cancer cell line. We found that expression of abundant CD95L-derived sequences as siRNAs were toxic to cells. In fact, CD95L-derived sequences also were enriched for toxicity when expressed as shRNAs [11], and this toxicity correlated well with the 6mer seed viability data [10].

In this study we determine the contribution of CD95L mRNA derived sRNAs to DISE, and the role of the miRNA biogenesis pathway in the processing and toxicity. We utilized Ago-RNA-pulldown small RNA-Seq combined with a SPOROS analysis (Ago-RP-Seq-SPOROS) to determine the contribution of CD95L mRNA derived sRNAs to the overall 6mer seed viability of the RISC in HCT116 cells lacking various components of the RNAi pathway. We found that the CD95L mRNA is processed into sRNAs that skew more toxic than reads derived from other highly expressed and processed mRNAs. We found that Dicer is not required for the processing of CD95L mRNA nor for the processing of mRNAs involved in translation. Interestingly, the role of Ago2 in mediating the toxic effects of CD95L mRNA expression appears to be cell type specific. While knockdown of Ago2 in ovarian [16] and breast cancer cell lines blocked toxicity by CD95L mRNA, knockdown or knockout of Ago2 did not rescue DISE induced by either CD95L mRNA or toxic seed containing shRNAs in the colon cancer cell line HCT116. However, the human genome encodes for four Argonaute proteins that may engage in RNAi. While Ago2 is the only Argonaute protein with slicer activity, all four Argonautes may silence expression of target mRNAs by perturbing translation [17]. Knockout of Argonautes 1–3, which are highest expressed in somatic cells, do not rescue CD95L mRNA toxicity. However, we found that Ago4 is significantly upregulated in these cells. Our analyses suggest that shifts in the 6mer seed viability of R-sRNAs (derived from CD95L and other genes) affect cell fate. Cell death is associated with an increased loading of sRNAs with toxic 6mer seeds, and decreased loading of sRNAs with non-toxic seeds. This is supported by the expression of various CD95L mutants. Expression of multiple toxic mutants induced a shift towards loading of toxic sRNAs, but a non-toxic mutant exhibited the opposite, increased loading of sRNAs with non-toxic 6mer seeds. Thus, our data suggests that CD95L mRNA can promote DISE directly, through loading of CD95L-derived sRNAs with toxic 6mer seeds, and indirectly, by promoting the loading of other toxic seed containing sRNAs into the RISC.

## Results

### RISC bound CD95L-derived sRNAs that contain the same sequence as shL3, a commercially available shRNA that is toxic to cancer cells

One commercial CD95L-derived shRNA that was found to be especially toxic was used in experiments that led to the discovery of DISE [11]. We referred to this sequence as shL3. Expression of shL3 in 293T cells lacking the shL3 target site resulted in the preferential loading of the 5-p arm or the sense strand of the shRNA [11]. In fact, there were 10 times more reads derived from the shL3 sense strand (5,039,726 sense reads to 477,308 antisense reads, Supplementary Fig. 1a). Of the five million shL3 sense reads, 85.6% shared the same 6mer seed sequence, ACTGGG (red box in Supplementary Fig. 1b). Based on our 6mer seed viability screens, we predict the average viability associated with this sRNA to be 37.6%. Alternative processing of this sequence (seen in 2.8% of cases) resulted in the generation of a seed that is even more toxic (GACTGG, seed viability 28%) (dark grey arrowhead on 5-p arm (Supplementary Fig. 1b). Conversely, the less abundant antisense strand was predicted to give rise to nontoxic sRNAs and the main species (full arrow head on 3-p arm, Supplementary Fig. 1b) is expected to have a 6mer seed viability of 80% (seed: ACAAAG, green box in Supplementary Fig. 1b). Thus, we conclude that the toxicity we observed from shL3 expression resulted from the activity of the much more abundant sense strand of the shL3-derived sequence.

**Fig. 1:**
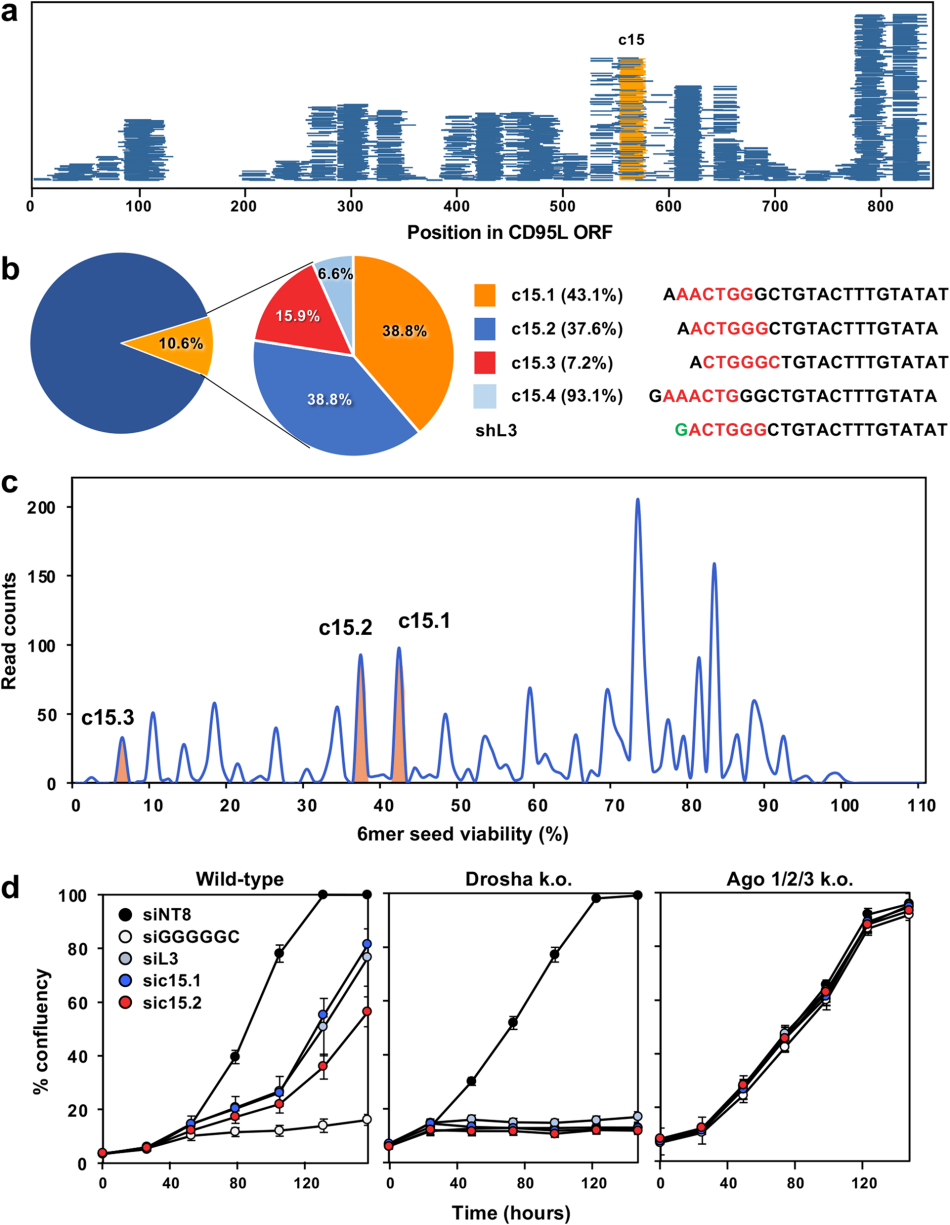
CD95L mRNA is processed to generate a sequence analogous to the toxic shRNA, shL3. **a** R-sRNAs derived from CD95L in HCT116 Drosha k.o. cells infected with pLenti-CD95L NP mapped along the ORF. Reads with the same sequence and 6mer seed as shL3 are indicated in orange. **b** *Left*, % of R-sRNAs derived from CD95L cluster 15. *Right*, breakdown of the percent of reads in cluster 15 (left) by 6mer seed (red lettering) and predicted 6mer seed viability (in parentheses). The most abundant sequences are numbered c15.1-c15.4. Green letter, vector derived. **c** Seed viability graph of CD95L-derived sequences in Drosha k.o. cells infected as in (**a**) with seed viability represented on the x-axis and normalized read counts on the y-axis. The three main read peaks that correspond to c15 are labeled in orange. **d** Confluency over time of wild-type, Drosha k.o., or Ago 1/2/3 k.o. cells transfected with 10 nM of control siRNA siNT8, the toxic human consensus containing siRNA siGGGGGC, the CD95L derived DISE inducing siRNA siL3, or siRNAs corresponding to c15.1 and c15.2. Data are representative of two independent experiments

CD95L mRNA is processed into R-sRNA that when expressed as siRNAs exert toxicity [16]. One of the most toxic of the abundant sequences aligned to cluster #15 (c15). We reanalyzed Ago-RP-Seq data from HCT116 Drosha k.o. cells expressing pLenti-CD95L NP (a mutant cDNA does not produce full length CD95L protein and has a point mutation that prevents binding of the truncated protein to CD95 [16]). We found that ~ 10% of reads derived from CD95L, those mapping to c15, contain the toxic shL3 sequence (Fig. 1a, b). The majority of these reads 77.6%) corresponded to two species (c15.1 and c15.2) with predicted toxic 6mer seeds. There were two more species with a small number of reads, one containing a highly toxic seed (c15.3), and one containing a nontoxic seed (c15.4).

Interestingly, one of the two predominant species in the RISC, c15.2, is nearly identical to shL3 (only position 1 differs, as in shL3 position 1 was derived from the pTIP vector sequence). Plotting the number of CD95L reads by their associated 6mer seed viability revealed that c15 is a substantial source of toxic sRNAs derived from CD95L (Fig. 1c). We confirmed that the sequence c15.2 exerts a negative growth effect through RNAi as HCT116 Drosha k.o. cells were more sensitive than wild-type (wt) cells to the toxicity when the sequence was introduced as an siRNA (Fig. 1d, left and center panel). This was in agreement with previous observations that Drosha k.o. cells are more sensitive to other toxic siRNAs, such as the FasL derived siRNA, siL3 [12], and an siRNA carrying the toxic consensus seed, GGGGGC [13] and likely due to the absence of most miRNAs protecting the RISC from uptake of sRNAs with toxic seeds [18]. Alternatively, Drosha k.o. cells could be hypersensitive to DISE due to a reduced ability to repair DNA [18] induced by toxic sRNAs [12]. In contrast, Ago 1/2/3 k.o. cells were completely resistant to c15.1 and c15.2 siRNA toxicity (Fig. 1d, right panel). These data suggest that CD95L can give rise to a R-sRNA that is almost identical to the commercial toxic CD95L-derived shRNA that resulted in the discovery of DISE.

### Processing of CD95L mRNA is independent of Dicer

Dicer has been reported to bind to mRNAs [19], and may even cleave mRNA substrates [20]. We therefore wondered whether Dicer was involved in the processing of CD95L mRNA. In our previous report, we found that CD95L-derived sRNAs mapped to regions of dsRNA in the predicted CD95L secondary structure, but detected some CD95L-derived sRNAs in Dicer k.o. cells using real-time quantitative (q)PCR; thus suggesting that Dicer may not be required for the processing of CD95L mRNA [16]. To test whether this finding could be generalized and to determine if either the pattern of CD95L processing or the loading of the derived sRNAs into the RISC was dependent on Dicer, we infected HCT116 wt, Drosha k.o. and Dicer k.o. cells with pLenti CD95L NP and sequenced the R-sRNAs. We observed greater toxicity in Drosha and Dicer k.o. cells expressing CD95L compared to wt cells, both by a greater reduction in cell growth and cell viability (Supplementary Fig. 2a, b). Interestingly, CD95L NP was also more highly expressed at the mRNA level (Supplementary Fig. 2c).

Analysis of R-sRNAs in HCT116 Dicer k.o. cells revealed that, like in Drosha k.o. cells, a variety of sRNAs other than miRNAs are found in the RISC (Supplementary Fig. 3a). This was expected as Dicer and Drosha are required for the biogenesis of most mature miRNAs. An analysis of the top five most abundant R-sRNAs in each genotype revealed that miRNAs dropped in abundance in k.o. cells (Supplementary Fig. 3a, b), and were replaced by many other sRNA species in Drosha k.o. and Dicer k.o. cells (Supplementary Fig. 3a, c, d). It was surprising that the predominant R-sRNAs in Dicer k.o. cells were derived from tRNA fragments (Supplementary Fig. 3a, d) as Dicer has been demonstrated to process some tRNAs [21]. This data likely reflects the fact that RNAs similar in structure and function can be processed via different mechanisms.

**Fig. 2:**
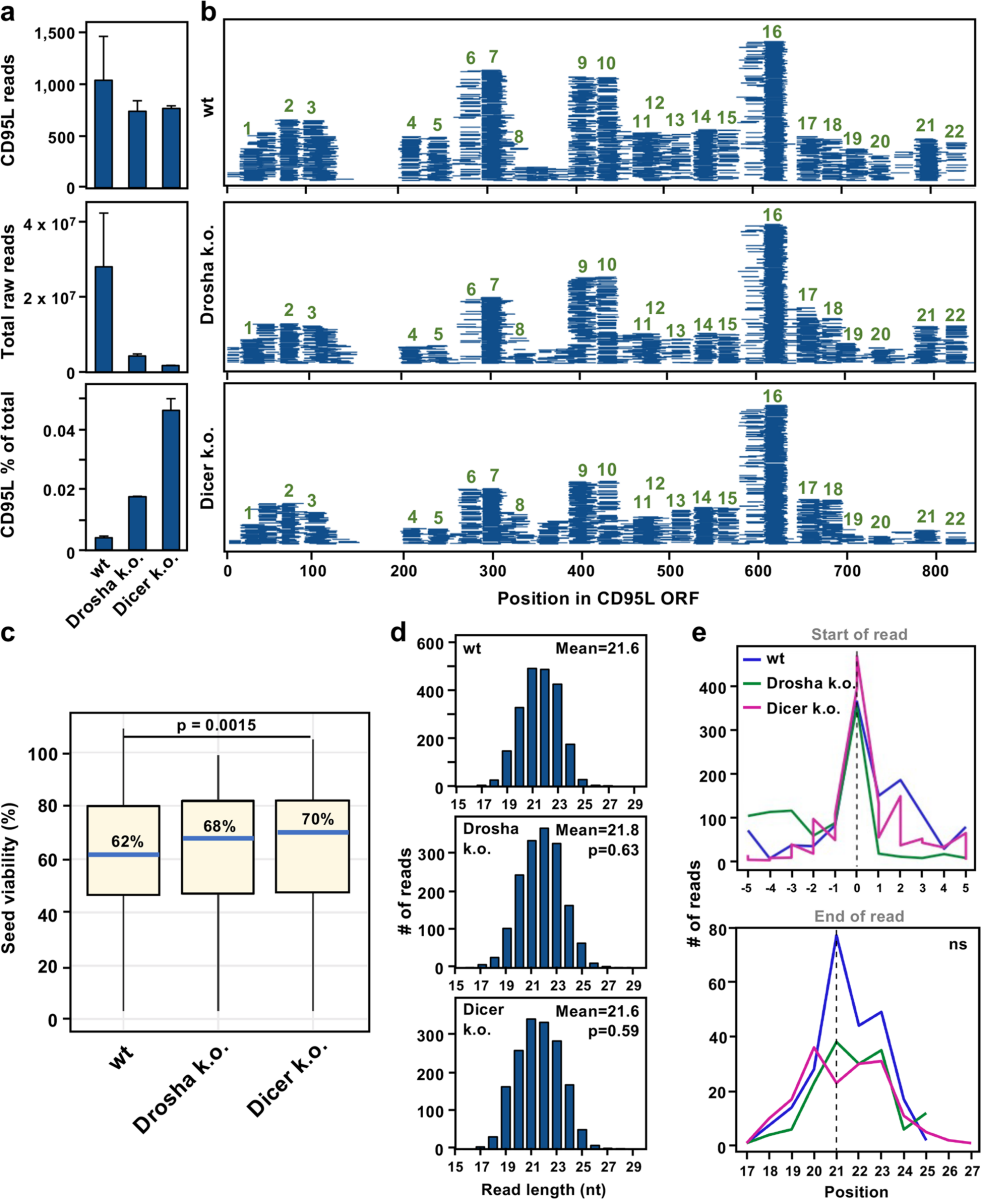
Loss of Dicer has no effect on the processing of CD95L-derived R-sRNAs. **a** Wild-type, Drosha k.o., or Dicer k.o. HCT116 cells were infected with pLenti-CD95L NP and Ago-RP-Seq analysis was performed 100 h after infection. *Top,* Raw R-sRNA counts aligning to CD95 NP. *Center,* the total raw R-sRNA reads sequenced per sample. *Bottom*, percentage of total raw R-sRNA reads derived from CD95L NP. Averages of two replicates are shown. Error bars represent the standard error of the mean. **b** Mapping of CD95L NP-derived sRNAs along the CD95L ORF. Each horizontal line represents one read. A combination of reads from both replicates are displayed. Stacks are numbered (in green as in [16]). **c** Box plots representing the distribution of the 6mer seed viability associated with CD95L-derived reads in the RISC in the samples in a. Blue lines and labels represent the median 6mer seed viability in each sample. Kruskal–Wallis test p-value is given. **d** Bar plots represent the read length distribution of all CD95L reads in the RISC. Significance was determined using unpaired T-tests. **e** The 10 most abundant stacks from b were analyzed for differential trimming. *Top*, the 5’ start position of the most abundant reads in each stack is indicated at 0. Reads with 5’ start sites either five nucleotides upstream or downstream were tallied. *Bottom*, the 3’ stop site of the 10 most abundant reads were tallied. Kruskal–Wallis *p*-value = 0.771 (ns, not significant)

To determine if Dicer is involved in the processing of CD95L mRNA, we identified all RISC-bound raw reads in our samples that uniquely mapped to the CD95L ORF (Fig. 2a, b). In this experiment, we found about the same number of reads derived from CD95L in all three genotypes (Fig. 2a, top). However, when we accounted for the number of reads in each sample (Fig. 2a, middle), we noticed that more of the RISC was occupied by CD95L-derived sRNA in Drosha k.o. cells (Fig. 2a, bottom), and surprisingly, an even higher percentage of the RISC was occupied by CD95L reads in Dicer k.o. cells. Examining the pattern of processing, we found no major differences between genotypes (Fig. 2b). Most reads seemed to be derived from similar regions of the mRNA. Analysis of the predicted 6mer seed viability of CD95L-derived reads revealed that Dicer k.o. cells had less toxic R-sRNAs (Fig. 2c), which could suggest some differential processing at the 5’ end of the sRNA. However, the lengths of CD95L reads did not vary significantly between genotypes (Fig. 2d). Closer examination of the 5’ start site and 3’ end site of the most abundant sRNAs revealed that the processing of CD95L was remarkably similar between genotypes. Thus, it is not likely that Drosha or Dicer function in the processing of CD95L mRNA.

### RISC bound reads of highly processed mRNAs in both Drosha and Dicer k.o. cells are derived from genes that function in protein translation

**Fig. 3:**
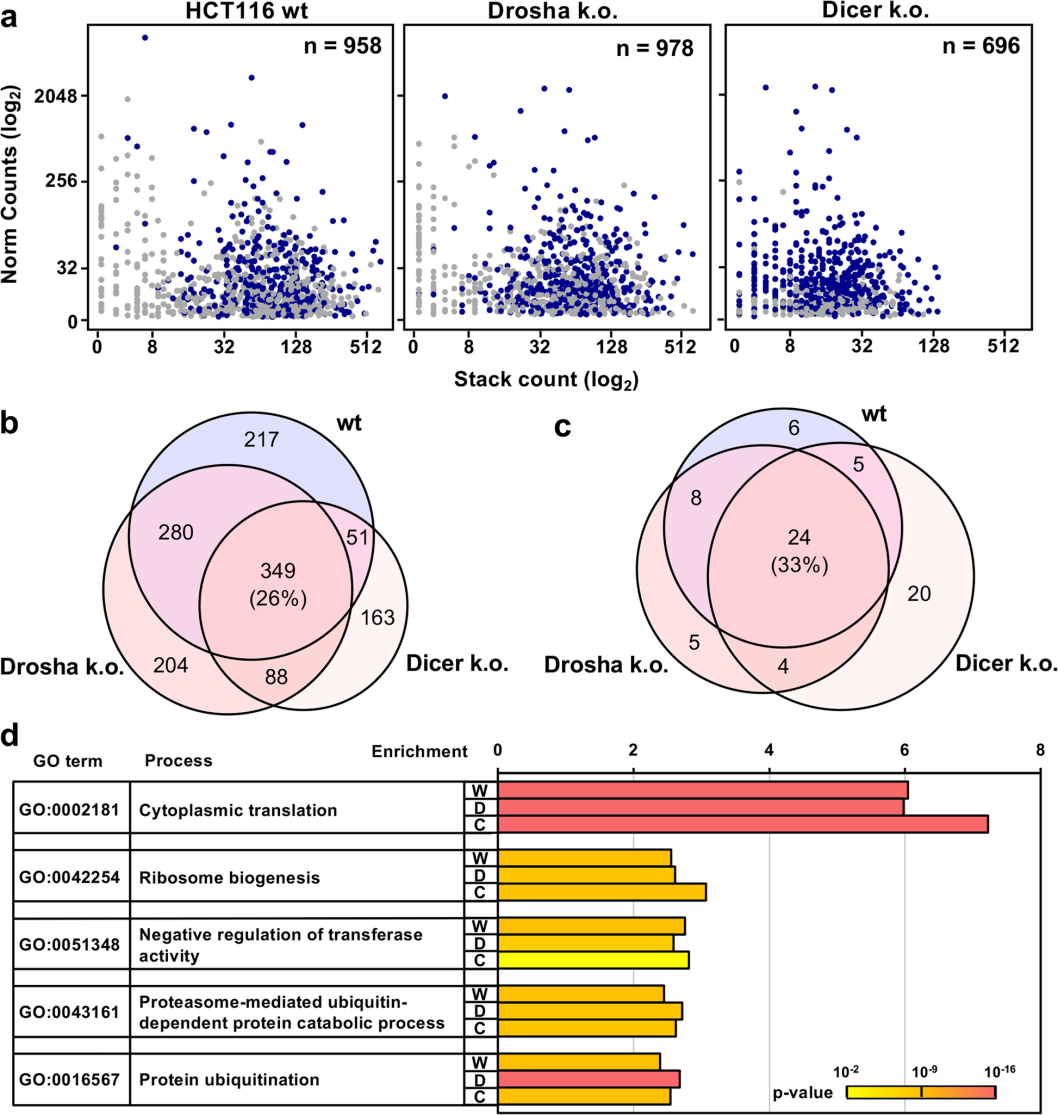
Protein-coding mRNAs involved in cytoplasmic protein translation are processed and loaded into the RISC independent of Dicer. **a** Dot plots representing processed mRNAs found in the RISC of HCT116 wt, Drosha k.o., and Dicer k.o. cells. Normalized gene expression is represented on the y-axis and stack count on the x-axis. A stack is defined as at least 10 raw reads mapping to a mRNA transcript with the same 5’ start site. Shared genes between all datasets are in dark blue. **b** Venn diagram displaying the overlap of genes processed in wt, Drosha k.o., and Dicer k.o. cells. **c** Overlap of significant GO terms (DAVID, Bonferroni adj. *p*-value < 0.05) enriched at least 1.5 fold in the three genotypes. **d** The top five enriched GO terms in all three genotypes. The Bonferroni corrected *p*-values are represented by color from *p* = 0.002 in yellow to *p* = 1.9 × 10^–16^ in pink. W, wt; D, Drosha k.o.; C, Dicer k.o

Previously we found that many mRNAs involved in translation and the cell cycle were processed in a similar manner as CD95L, particularly in Drosha k.o. cells [16]. To confirm this finding and determine if these mRNAs were processed in both Drosha and Dicer k.o. cells, all R-sRNAs from cells infected with pLenti CD95L NP were aligned to the human genome and sRNA reads mapping uniquely to protein coding genes were plotted. As a way of measuring processing, we defined a stack as at least 10 raw reads mapping with the same 5’ start site to a gene. Genes were considered processed if there were three or more stacks mapping to a gene and at least 10 normalized reads. These criteria were met by many hundreds of genes in each genotype (Fig. 3a). Of these, 349 (26%) processed mRNAs were shared (Fig. 3b). To determine if the processed mRNAs were derived from genes with similar functions, we performed a gene ontology (GO) analysis, and found that 33% of enriched GO terms were shared (Fig. 3c). Consistent with our previous report, genes involved in protein translation were highly enriched in both Drosha and Dicer k.o. cells (Fig. 3d). Other GO terms that were shared suggest that genes involved in proteasome and ubiquitin dependent protein catabolism are also selectively processed.

Examining the pattern of processing of highly processed mRNA transcripts revealed subtle differences in the nature of the reads loaded into the RISC of HCT116 wt, Drosha, and Dicer k.o. cells (Supplementary Fig. 4). While processing of FAT1 was very similar between genotypes (Supplementary Fig. 4a), ACTG1 exhibited some differences in processing and/or loading of reads into the RISC (Supplementary Fig. 4b). In other genes certain stacks abundant in Drosha k.o. and wt cells were almost absent in Dicer k.o. cells (red arrows in Supplementary Fig. 4c, d). Nevertheless, our data do not support a role for Dicer in the processing and loading of these sequences. Mapping abundant R-sRNAs derived from EEF1A1 to its predicted secondary structure revealed that these sequences do not map to stem loop structures or regions of dsRNA, as might be expected of potential Dicer substrates (data not shown). Thus, it is not likely that Dicer is a mediator of the processing of these mRNAs. However, there remains a possibility that Dicer could play a role in the loading of mRNA-derived sRNAs into the RISC.

### In HCT116 cells the toxicity of neither CD95L mRNA nor CD95L-derived shRNAs depends on Ago2

**Fig. 4:**
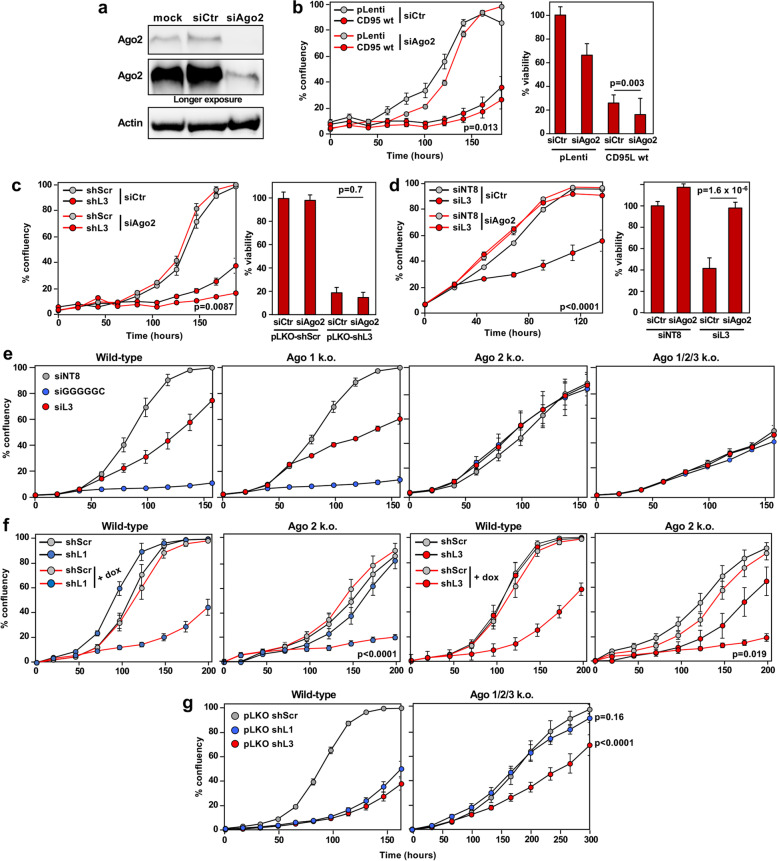
Ago2 is not required for toxicity of CD95L mRNA or DISE-inducing si-/shRNAs in HCT116 cells. **a** Western blot analysis of Ago2 expression in HCT116 Drosha CD95 d.k.o. c12 mock transfected or transfected with 25 nM siAgo2 or scrambled control (siCtr) siRNA SmartPools. **b** *Left*, Percent confluency over time of HCT116 Drosha CD95 d.k.o. c12 transfected with either 25 nM siAgo2 or siCtr siRNAs, and subsequently infected with pLenti or pLenti-CD95L. *Right*, relative cell viability at 120 h. **c** *Left*, percent confluency over time of HCT116 Drosha CD95 d.k.o. c12 transfected with either 25 nM siAgo2 or siCtr siRNAs, and subsequently infected with pLKO-shScr or pLKO-shL3 (left). *Right*, relative cell viability at 120 h. **d** *Left*, Percent confluency over time of HCT116 Drosha CD95 d.k.o. c12 transfected with either 25 nM siAgo2 or siCtr siRNAs, and subsequently transfected with 10 nM siL3 or siNT8 non-targeting control. *Right*, relative cell viability at 96 h. **e** Percent confluency over time of HCT116 wt (left), Ago1 k.o. (center left), Ago2 k.o. (center right), and Ago 1/2/3 k.o. cells (right) transfected with 10 nM siGGGGGC, siL3, or siNT8 control. **f** Percent confluency over time of HCT116 wt and Ago2 k.o. cells stably expressing either doxycycline (dox) inducible pTIP-shScr, pTIP-shL1, or pTIP-shL3. Cells were left untreated or treated with 100 ng/ml dox at 0 h. **g** Percent confluency over time of HCT116 wt (left) and Ago 1/2/3 k.o. cells (right) infected with pLKO-shL1, pLKO-shL3 or pLKO-shScr. Error bars in b-d represent the standard deviation of quadruplicates. Statistical significance was evaluated by Two-way analysis of variance (ANOVA) in b (left), c (left), d (left), f, and g; and by Student's T-Test in b (right), c (right), and d (right)

We previously observed that Ago2 was required for CD95L toxicity in HeyA8 CD95 k.o. cells [16]. Thus, we wondered whether the catalytic activity of Ago2 could be involved in the trimming of CD95L reads in addition to mediating CD95L toxicity through RNAi. To test this hypothesis, we generated HCT116 Drosha CD95 double knock-out (d.k.o.) cells. These cells would allow for analysis of wt CD95L mRNA processing while preventing the apoptosis inducing activity of any CD95L protein expression. Three homozygous CD95 mutant clones were isolated (Supplementary Fig. 5a), all three did not express any detectable surface CD95 (Supplementary Fig. 5b) and retained sensitivity to pLKO-shL3 (Supplementary Fig. 5c, d). Efficient Ago2 knockdown was achieved in HCT116 Drosha CD95 d.k.o. clone 12 (Fig. 4a). However, this did not rescue CD95L toxicity (Fig. 4b). This was not a clonal effect as Ago2 k.d. also did not rescue toxicity induced by CD95L NP in HCT116 wt cells or parental Drosha k.o. cells (Supplementary Fig. 6a). It is possible that the role of Ago2 in CD95L toxicity is cell type specific; Knockdown of Ago2 was able to rescue toxicity in HeyA8 cells [16] and in CD95 k.o. MCF7 cells (Supplementary Fig. 6b).

Our data led us to wonder if knockdown of Ago2 was sufficient to block RNAi in HCT116 cells. Interestingly, knockdown of Ago2 in the same d.k.o. clone was not sufficient to block DISE induced by shL3 (Fig. 4c). This was surprising, as Ago2 is thought to be the primary mediator of RNAi in all cells. To determine if this effect was specific to shL3, we transfected toxic DISE-inducing siRNAs into these cells. Knockdown of Ago2 did block the toxicity of siL3 [11] (Fig. 4d). It is possible that knockdown of Ago2 was insufficient to rescue the toxicity induced by constitutively expressed sequences. Thus, we tested Ago1, Ago2 and Ago 1/2/3 k.o. HCT116 cells to determine if cells completely devoid of these Argonaute proteins were resistant to the toxic effects of DISE-inducing si- or shRNAs. Knockout of Ago2 and triple k.o. of Ago 1/2/3 blocked the toxicity exerted by the siRNAs siL3 and siGGGGGC [13], while Ago1 k.o. cells remained sensitive (Fig. 4e). However, Ago2 k.o. cells retained sensitivity to the DISE inducing shRNAs, shL1 and shL3 (Fig. 4f). It is possible that these shRNAs may exert toxicity through other Ago proteins. Interestingly, knockout of Ago 1/2/3 in HCT116 cells rescued shL1 toxicity but the cells remained somewhat sensitive to shL3 (Fig. 4g). This led us to wonder if the remaining Ago protein present in HCT116 cells, Ago4, may also mediate DISE.

### CD95L mRNA is toxic to HCT116 Ago1/2/3 k.o. cells

**Fig. 5:**
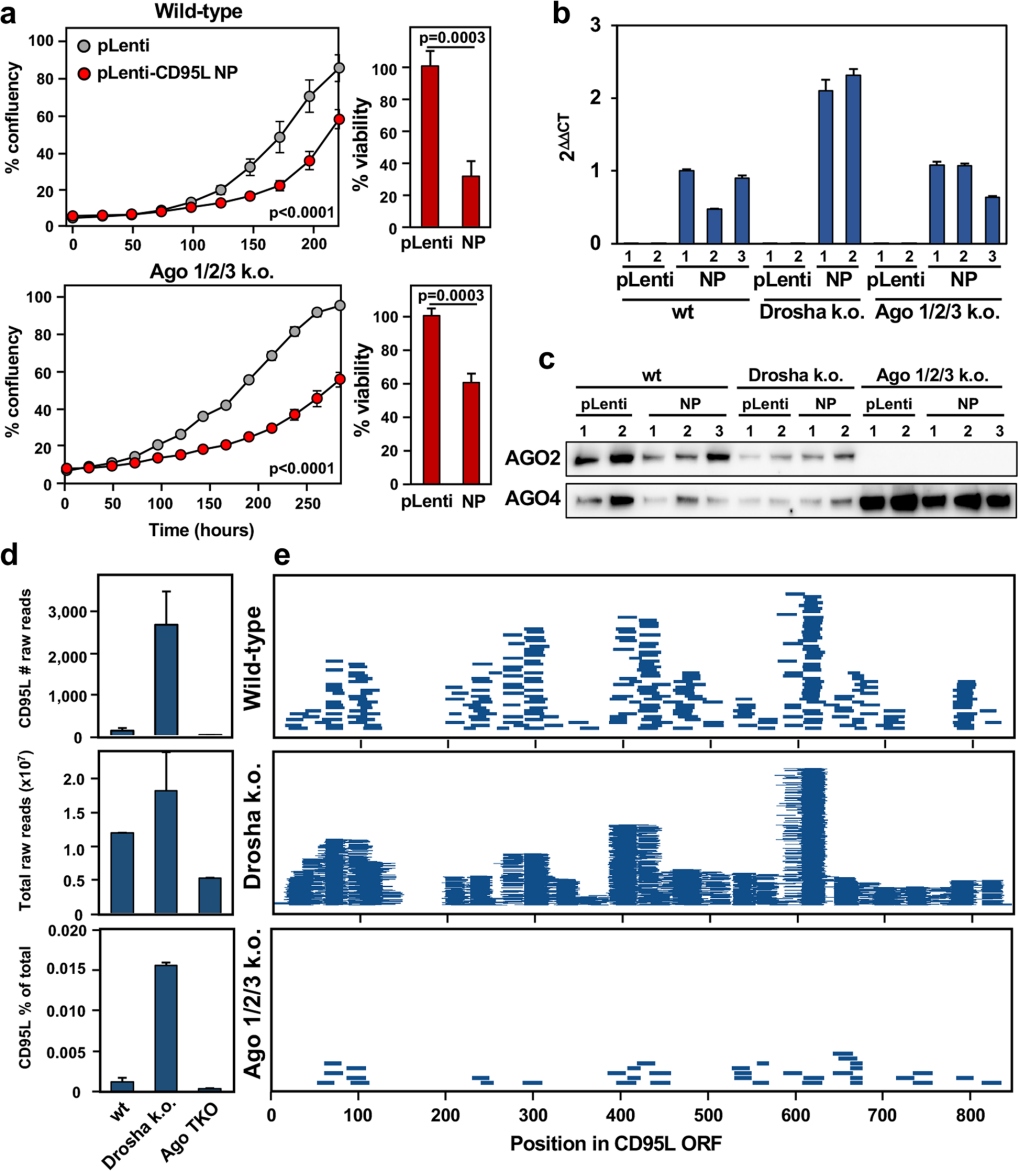
CD95L NP is toxic to HCT116 Ago 1/2/3 k.o. cells. **a** *Left*, Percent confluency over time in HCT116 wt (top) and Ago 1/2/3 k.o. cells (bottom) expressing pLenti empty vector control (pLenti) or pLenti CD95L NP (NP). Statistical significance was evaluated two-way ANOVA. *Right*, Relative cell viability at 144 h. Error bars represent the standard deviation of triplicates. Statistical significance was determined by unpaired T-test. **b** Real-time qPCR analysis of CD95L expression in samples collected for Ago pulldown experiments, normalized to GAPDH. Bars represent the mean with standard deviation of triplicates. **c** Western blot analysis of Ago protein pulldown by the T6B peptide in HCT116 wt, Drosha k.o., and Ago 1/2/3 k.o. cells. **d** Raw counts of R-sRNAs aligning to CD95L NP (top). The total raw R-sRNA reads sequenced per sample (center). The percentage of total raw R-sRNA reads that are derived from pLenti-CD95L NP (bottom). The average of two replicates are shown. Error bars represent the standard error of the mean. **e** Mapping of CD95L NP-derived R-sRNAs along the CD95L ORF in the indicated genotypes. Each horizontal line represents one read. Reads from both replicates are displayed

The association of the four Argonaute proteins in human cells with miRNAs has been found to be largely redundant [17, 22]. Due to this observation, the fact that Ago4 lacks endonuclease activity [23–25], and is often expressed at low levels in wt cells [17, 26], it is unclear if Ago4 mediates canonical RNAi or if it exerts unique cellular functions. Our data did present the possibility that Ago4 may be functional in mediating DISE in Ago 1/2/3 k.o. cells. We observed that Ago 1/2/3 k.o. cells were moderately sensitive to expression of pLenti-CD95L NP, similar to shL3 (Fig. 5a). Expression of the mRNA was similar between HCT116 wt and Ago 1/2/3 k.o. cells but increased in Drosha k.o. cells (Fig. 5b and Supplementary Fig. 7b). To exclude the possibility that CD95L expression would affect the expression of housekeeping genes used to normalize the qPCR analysis, we performed this analysis twice, once normalized to GAPDH, which was slightly downregulated in our previous RNA-Seq analysis of CD95 k.o. HeyA8 cells 50 h after expression of CD95L [16], and once normalized to β-actin, which was slightly upregulated in CD95L NP expressing cells (Supplementary Fig. 7a). To determine if CD95L could still be exerting toxicity through CD95L-derived sequences in the RISC, we performed Ago-RP-Seq. In the absence of Ago 1–3, Ago4 expression was substantially upregulated resulting in the dramatically increased pulldown of Ago4 in these k.o. cells observed by western blotting (Fig. 5c). Upon analysis of the Ago4 bound sRNAs, we found very few CD95L-derived reads in the RISC (Fig. 5d), an observation seemingly inconsistent with RNAi being involved in the toxicity.

### CD95L-derived reads skew more toxic than reads derived from other mRNAs but the RISC favors nontoxic reads

While our data demonstrated that CD95L-derived sRNAs can exert toxicity in cells, it was unclear if this was a characteristic specific to CD95L mRNA or if it could be a general property of processed mRNAs [11, 16]. Because many of these genes are involved in protein translation and cell cycle control, it might be surprising if these genes that drive cell proliferation negatively regulated cell survival through their mRNA. We identified the top 10 processed mRNAs by ranking coding genes by expression and processing, and selected genes abundant in both the total sRNA and the RISC. These genes, in addition to CD95L, all had greater than 10 stacks in the RISC (Supplementary Fig. 8a) and in the total sRNA (Supplementary Fig. 8b). Applying the 6mer seed viability data to all sRNAs 18–25 nts long, we found that CD95L-derived reads skewed more toxic than those derived from the top 10 processed mRNAs (Supplementary Fig. 8c). This was not merely a feature of exogenously expressed CD95L as endogenous CD95L-derived reads in cells expressing pLenti empty vector controls were also similarly toxic (Supplementary Fig. 8c, right panel), although present at much lower levels (Supplementary Fig. 8d). A breakdown of the median 6mer seed viability of total cellular sRNAs by gene predicted that CD95L-derived sRNAs were more likely to exhibit toxicity through RNAi (Supplementary Fig. 8e). However, R-sRNAs derived from CD95L exhibited only a slightly lower median 6mer seed viability distribution than the R-sRNAs derived from the top 10 genes (Supplementary Fig. 8f). The median 6mer seed viability of CD95L-derived reads was 70%, while that of all Top 10 genes in aggregate was 73%. This contrasted to the median seed viability of all R-sRNAs which was 26% (Supplementary Fig. 8f, right panel). We conclude that highly expressed processed protein coding genes are not favored to produce sRNAs that will exert toxicity through RNAi. Interestingly, while CD95L mRNA is a source of toxic sRNA, this analysis suggested that the majority of CD95L-derived sRNA loaded into the RISC were not likely to exert toxicity. This analysis also suggested that CD95L mRNA expression may affect cell fate by changing the composition of R-sRNAs derived from other sources.

### Exploring the sequence determinants of CD95L toxicity

**Fig. 6:**
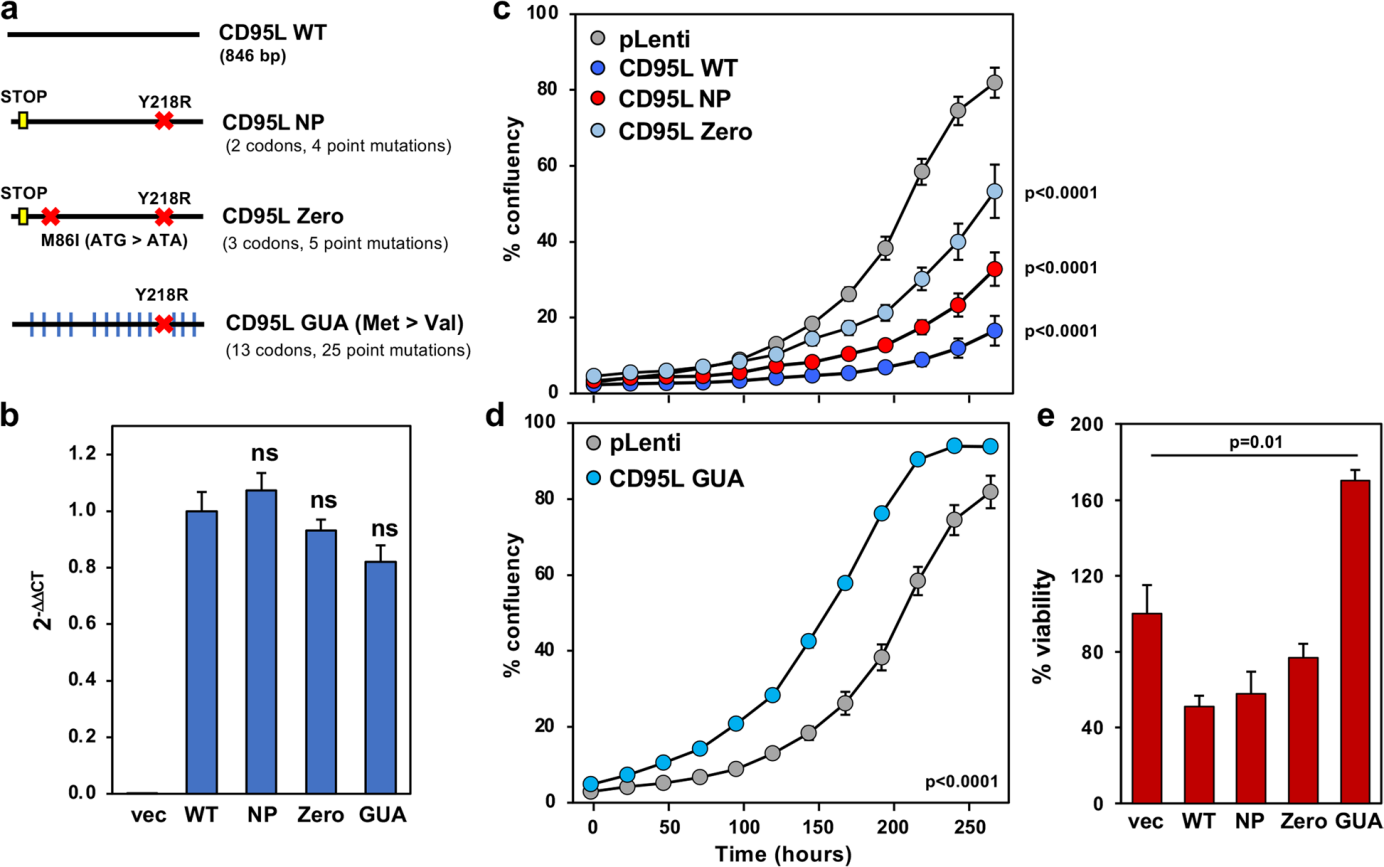
The mutant CD95L GUA is not toxic to cells. **a** Scheme representing the various CD95L mutants. A yellow box represents a mutation to a stop codon (UGA), an X indicates a point mutation, and a blue vertical line indicates mutation of an in-frame start codon (AUG) to valine (GUA). **b** Real-time qPCR analysis of CD95L mRNA expression in HCT116 Drosha CD95 d.k.o. c12 100 h after infection with the CD95L mutants pLenti empty vector (vec), wild-type CD95L (WT), CD95L NP (NP), CD95L Zero (Zero), CD95L GUA (GUA). Expression was normalized to GAPDH and pLenti wild-type CD95L. Bars represent the standard deviation of triplicates. Statistical significance was evaluated by unpaired T-test. ns, not significant. Data is representative of two independent experiments. **c** Percent confluency over time of HCT116 Drosha CD95 d.k.o. c12 infected with pLenti empty vector, pLenti-CD95L WT, pLenti-CD95L NP, pLenti-CD95L Zero, and **d** pLenti-CD95L GUA. Error bars represent the standard error of triplicates. Two-way ANOVA was utilized to assess statistical significance. **e** Relative cell viability at 120 h. Error bars represent the standard deviation of quadruplicates. Statistical significance was determined by unpaired T-test

One of the main barriers to studying the toxic effects of CD95L mRNA is that the protein is a well characterized inducer of extrinsic apoptosis. To separate the effects of the mRNA from the protein, we previously generated various CD95L mutants. CD95L NP (Fig. 6a) exerts toxicity through a caspase-independent mechanism [16]. CD95L Zero is a further mutated version of CD95L NP with an additional mutation in the first alternative start codon (AUG > AUA) (Fig. 6a). This blocked CD95L protein expression but retained toxicity [16]. We also hypermutated the CD95L sequence, introducing 303 synonymous mutations in CD95L SIL [16]. This mutant was still toxic. The only mutant we found to be non-toxic had all in-frame start codons mutated to stop codons. However, expression of this mutant was significantly reduced (data not shown). We suspect that introduction of premature stop codons activated nonsense mediated decay (NMD) [27]. To avoid activating NMD, we mutated all in-frame start codons from AUG to GUA (Val) (Fig. 6a). Expression of this mutant, CD95L GUA produced mRNA at similar levels as wt CD95L and the other mutants (Fig. 6b and Supplementary Fig. 7c), but in contrast to these constructs (Fig. 6c) CD95L GUA was nontoxic, and even appeared to promote cell growth (Fig. 6d, e).

**Fig. 7:**
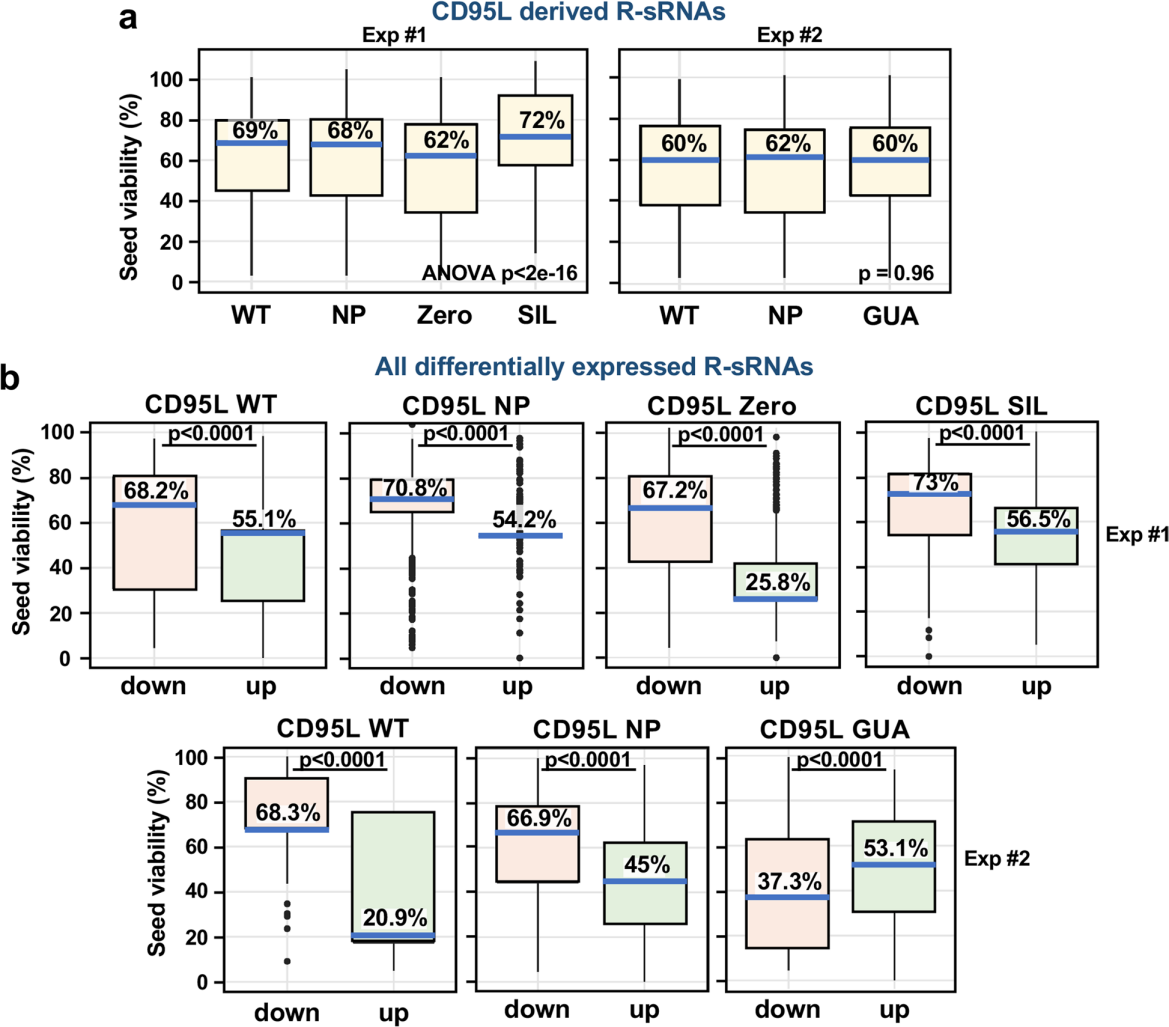
Shifts in the 6mer seed viability of R-sRNAs predict the toxic activity of mutant CD95L. **a** Boxplots with 6mer seed viabilities of the CD95L-derived sRNAs in the RISC. Reads from various CD95L mutants were compared. Data presents two independent experiments, experiment 1 (left) and experiment 2 (right). Kruskal–Wallis test *left: p* < 2 × 10^–16^ (the lower p-value threshold in R) and *right*: *p* = 0.951. **b** The 6mer seed viability of all differentially expressed R-sRNAs upon expression of various CD95L mutants. Only differentially expressed reads with an adjusted *p*-value < 0.05 are represented with the 6mer seed viability of down regulated reads in red and upregulated reads in green. Two independent experiments are represented with experiment one (top) and experiment two (bottom). Blue horizontal lines represent the median 6mer seed viability (% seed viability is given). Kruskal–Wallis test *p*-values are given

To determine if CD95L mRNA processing and loading into the RISC differed between the various mutants, we performed an Ago-RP-Seq analysis. The expression of CD95L-derived reads and their contribution to the RISC-bound sRNA population was quite similar between mutants in the d.k.o. cells (Supplementary Fig. 9a) and so was the average sRNA read length (Supplementary Fig. 9b). Read stacks were also quite similar among the mutants with the exception of the SIL mutant (Supplementary Fig. 9c). Stacks were often located close to stem regions (Supplementary Fig. 9c, secondary structure prediction shown below each stack plot) suggesting that whatever is processing these mRNAs exhibits a preference for cleavage at regions of double-stranded RNA. All mutants showed a glaring lack of reads in the region spanning positions 133–200. In each case this region was enriched in cytosines (corresponding to the proline richness of the corresponding protein stretch) suggesting that C-rich regions either are not processed or not loaded into the RISC. Similarity between the mutants was also reflected in the average seed viability of all CD95L-derived reads in the RISC (Fig. 7a). The seed viability associated with each CD95L mutant was quite similar, although R-sRNAs derived from the CD95L SIL mutant were slightly less toxic. These data thus indicated no correlation between the seed viability of CD95L-derived R-sRNAs and their effect on cell viability.

We recently provided evidence in a number of models that suggests that it is the balance of all RISC bound sRNAs with toxic versus nontoxic seeds that can determine cell survival [15, 28, 29]. Using SPOROS we analyzed the 6mer seed viability of reads that were significantly enriched or depleted (adjusted *p*-value < 0.05) in the RISC of d.k.o cells infected with the various CD95L mutants. In samples expressing toxic CD95L mutants, the 6mer seed viability of the RISC shifted towards selective loading of more toxic sRNAs; Toxic R-sRNAs were significantly enriched and non-toxic R-sRNAs were downregulated (Fig. 7b). In contrast, the non-toxic CD95L GUA mutant, caused a shift towards loading of non-toxic sRNAs (Fig. 7b). These data suggest that a combination of CD95L-derived reads and reads derived from other genes regulate cell fate upon introduction of CD95L mRNA.

## Discussion

We have reported that CD95L can kill cells independent of binding to its receptor, CD95, and provided evidence that the mRNA kills cells through an RNAi-based mechanism similar to DISE [16]. We found that CD95L mRNA processing results in the production of the same shRNA sequence that led to the discovery of DISE [11]. This finding fueled further exploration of the role of the RNAi biogenesis machinery and Argonaute proteins in CD95L mRNA processing and toxicity.

Our recent development of the Ago-RP-Seq-SPOROS pipeline [14] enabled us to explore the connection between sRNAs in the RISC and cell death in a standardized way. By applying data from 6mer seed viability screens, we connect R-sRNAs to phenotypic outcomes. Our Ago-RP-Seq-SPOROS analyses agreed with our experimental observations that expression of CD95L-derived sRNAs as si-/shRNAs exert high levels of toxicity in cells. Further, we found that CD95L processing generates sRNAs that are more likely to exert toxicity than sRNAs derived from other highly expressed, processed, and RISC loaded endogenous mRNAs. Thus, we conclude that CD95L mRNA induces toxicity directly, through the activity of R-sRNA derived from the processing of its mRNA. However, our data did not exclude that possibility that other endogenous R-sRNAs may contribute to this effect. We recently showed that shifts in the balance of R-sRNAs with toxic versus non-toxic 6mer seeds can effect phenotypic outcomes in therapy resistant ovarian cancer [15], in Alzheimer’s disease [28] and in HIV-1 infected cells [29]. Here we report that similar shifts in the balance of toxic versus non-toxic sRNAs in the RISC predict cell fate decisions. Expression of a number of toxic CD95L constructs induced shifts towards increased RISC loading of toxic sRNAs and decreased loading of non-toxic sRNAs. Conversely, expression of the non-toxic CD95L mutant, CD95L GUA, induced a shift towards loading of nontoxic sRNAs into the RISC. Thus, our data indicate that CD95L mRNA induces changes in RISC loading of sRNA that promote toxicity.

We observed that knockdown of Ago2 rescued CD95L mRNA toxicity in an ovarian cancer cell line. Thus, we assumed that Ago2, the only Argonaute protein with catalytic activity, was the primary mediator of CD95L mRNA toxicity. However, our current observations suggest that this dependence on Ago2 may be cell type specific. The mammalian genome encodes four Argonaute proteins. Ago2 is the only protein with slicer activity, that is capable of cleaving target mRNA [24, 25] and promoting miRNA biogenesis [30, 31]. However, target mRNA cleavage is not required for gene silencing, and all Argonaute proteins may mediate RNAi somewhat redundantly [17, 32], although some studies have ascribed specific activities to individual Argonaute proteins [33–35]. Knockdown of Ago2 in a MCF7 CD95 k.o. breast cancer cell line attenuated CD95L mRNA toxicity, supporting our observations in HeyA8 CD95 k.o. cells. However, knockdown of Ago2 did not rescue CD95L toxicity in wt, Drosha k.o. or Drosha CD95 d.k.o. HCT116 cells. Interestingly, neither knockdown nor knockout of Ago2 in HCT116 Drosha CD95 d.k.o. cells rescued toxicity exerted by DISE-inducing shRNAs. Only the activity of toxic DISE-inducing siRNAs was rescued by knockdown and knockout of Ago2. This may suggest that the kinetics or level of shRNA expression could overcome the loss of Ago2. Perhaps the constitutive expression of an shRNA increases the likelihood it will act as a guide RNA in the catalytic deficient Ago proteins 1, 3, or 4. Because DISE results from seed-based targeting similar to miRNA targeting, we expect that catalytic-deficient Argonaute proteins could mediate DISE through seed-based targeting of survival genes.

Our data suggest that the HCT116 colon cancer cell line exhibits a special regulation of the RNAi pathway. These cells are uniquely amenable to genetic deletion of RNAi pathway components, which could be due to their ability to genetically compensate. For example, these cells drastically upregulate Ago4 in response to the deletion of Ago 1–3 [29]. We hypothesize that this genetic robustness may underlie the differential effects of Ago2 knockdown on CD95L toxicity. HCT116 cells may be better able to functionally compensate for a loss of Argonaute expression than HeyA8 or MCF7 cells. The loss of Ago 1–3 in our report did result in decreased loading of CD95L-derived reads into the RISC, but despite this reduction in RISC-bound CD95L reads cells remained sensitive to CD95L mRNA toxicity. While the number of CD95L-derived R-sRNAs pulled down with Ago4 in the Ago 1/2/3 triple knock-out cells was very small, it was not smaller than the number of CD95L-derived R-sRNAs pulled down in CD95L infected HeyA8 cells [16]. The same HeyA8 cells were used to establish that CD95L NP expression promotes death through RNAi. Overall, our data suggest that death may result not only from loading of toxic CD95L-derived reads into the RISC, but also from increased loading of other sRNAs with toxic 6mer seed sequences.

A major unanswered question remains: what enzymes process mRNAs to produce R-sRNAs? The observation that CD95L-derived sRNAs map to regions of dsRNA in the predicted secondary structure of CD95L mRNA led us to hypothesize that Dicer could mediate processing. We performed Ago-RP-Seq in HCT116 Dicer k.o. cells expressing pLenti-CD95L NP and found no difference in the processing or loading of R-sRNAs. In fact, virtually the same CD95L-derived sRNAs found in the RISC of Drosha k.o. cells appeared to also be more abundant in the RISC of Dicer k.o. cells. Likewise, endogenous processed mRNAs, especially mRNAs involved in protein translation were also processed and loaded into the RISC in the absence of Dicer. Thus, the endonucleases that produce mature miRNAs, Drosha and Dicer, are not involved in the generation of R-sRNAs derived from protein coding mRNAs. Our data are consistent with previous reports in which we showed a strong sensitization of cells lacking any of the miRNA processing genes (e.g., Drosha or Dicer) to DISE inducing stimuli [10, 11, 15, 16, 28, 29] but add another layer by suggesting a connection between RNAi and protein translation.

Multiple mechanisms of RNA degradation are coordinated at ribosomes such as nonsense-mediated decay, non-stop decay, and no-go decay [36]. These pathways degrade mRNAs that lack stop codons, contain premature stop codons, or are bound by stalled ribosomes. Our observations involving the expression of various CD95L mutants suggest that CD95L processing and toxicity could be regulated at ribosomes. In HCT116 cells we could not rescue toxicity by manipulating the RNAi pathway. Only changes to the sequence of CD95L prevented toxicity. While introduction of 303 synonymous point mutations had no effect on toxicity, it did significantly alter the pattern of mRNA processing. The only change to the CD95L sequence that rescued toxicity was the mutation of all in-frame start codons to valines in the mutant CD95L GUA. While these mutations did not drastically alter the pattern of mRNA processing, it is likely that they would change the binding and translation of this transcript by ribosomes. Stalled ribosomes and ribosome collisions on mRNA transcripts have been shown to activate endonucleolytic cleavage of mRNA transcripts and could be involved in initiating the processing of CD95L mRNA and other mRNAs in the RISC. In fact, RNAi may trigger ribosome stalling, collisions, and transcript processing, especially in the absence of the ribosome rescue factor PELO and the RNA degrading ski complex, as was shown in in *C. elegans* [37]. Thus, RNAi could even initiate such processing of an mRNA. It has been demonstrated that phased cleavage products generated at stalled ribosomes persist in cells [38]. Thus far, the endonuclease that generates these persistent cleavage products through riboythrypsis, has yet to be identified. The endonucleases NONU-1 and Cue2 were shown to function in translational surveillance, and to cleave mRNA upstream of stalled ribosomes in no-go decay in *C. elegans* and yeast, respectively [39, 40]. However, the human orthologue, N4BP, has yet to be implicated in mRNA processing. Mounting evidence demonstrates that RNA degradation pathways function redundantly, and that the activity of some pathways may only be observable when another RNA degradation pathway is perturbed [41], and/or in the absence of other quality control pathways, such as the Ribosome Quality Control Trigger complex [40]. Thus, identification of the endonuclease involved in the processing of R-sRNAs-derived from mRNAs may be difficult using genetic tools like loss of function genetic screens.

RNA degradation pathways are likely to be intricately connected to RNAi in cells. RNAi has been shown to promote mRNA transcript degradation by the decapping complex and the deadenylase complex [42, 43]. Increasing evidence supports a role for the non-stop translation surveillance pathway in the clearance of ribosomes from targeted transcripts and subsequent mRNA degradation [44–46]. Interestingly, this process appears to amplify the silencing activity of the RISC [37]. One could speculate a relationship may exist between the activation of RNA surveillance pathways and RNAi to prevent the translation of toxic proteins or peptides. Future work should determine the degree to which CD95L and other processed mRNAs are regulated by such RNA surveillance pathways. The link between known targets of no-go decay and RNAi could also be explored. If RNAi promotes more ribosome stalling and no-go decay, R-sRNAs derived from mRNAs may further promote RNAi, or they could function as inhibitors of canonical RNAi. The idea that mRNA degradation products are not merely recycled but could in turn regulate gene expression is intriguing and could contribute to our understanding of the role of RNA in the regulation of gene expression at homeostasis and in the context of disease.

## Materials and methods

### Reagents and antibodies

Reagents used were puromycin (Sigma, #P9620), doxycycline (Sigma, #9891), leucine-zipper tagged (Lz)CD95L described in [47], Lipofectamine RNAiMAX (ThermoFisher Scientific, #13,778,150), Lipofectamine 2000 (ThermoFisher Scientific, #11,668,027), polybrene (Sigma, #H9268). The following antibodies were used: PE conjugated-anti-CD95 (eBioscience, cat #12–0951-83; RRID: AB_465789); PE conjugated Isotype control (eBioscience, cat #12–4714-82; RRID: AB_470060); anti-β-actin antibody (Santa Cruz, #sc-47778, RRID: AB_626632); anti-AGO2 (Abcam, #32,381 AB_867543), anti-AGO4 (Cell signaling #6913, RRID: AB_10828811).

### Cell culture

293T cells were cultured in DMEM medium (Corning #10–013-CM) with 10% Serum Plus II Medium Supplement (Sigma-Aldrich #14009C). Generation of MCF7 CD95 k.o. clone was described in [16]. MCF7 CD95 k.o. cells were cultured in RPMI 1640 medium (Corning #10–040 CM) supplemented with 10% Serum Plus II Medium Supplement (Sigma-Aldrich #14009C) and 1% penicillin/streptomycin (Mediatech Inc). The following HCT116 cells were purchased from the Korean Collection for Type Cultures (KCTC): HCT116 wild-type (KCTC, cat #HC19023), HCT116 Drosha k.o. clone #40 (KCTC, cat #HC19020), HCT116 Dicer k.o. clone #43 (KCTC, cat #HC19023). The HCT116 Ago2 k.o. cells [48] were a kind gift from Joshua T. Mendell (UT Southwestern). The HCT116 Ago1 k.o. and HCT116 Ago 1/2/3 k.o. cells [26] were provided by David Corey (UT Southwestern). All HCT116 cells and knock-out clones were cultured in McCoy’s 5A medium (ATCC #30–2007) with 10% Serum Plus II Medium Supplement (Sigma-Aldrich #14009C). Cells were cultured at 37 °C, 5% CO_2_ were tested for mycoplasma using PlasmoTest (Invitrogen). Before use the Serum Plus II Medium Supplement was heat inactivated by incubation at 56 °C for 30 min.

### CRISPR-Cas9 deletion of CD95

HCT116 Drosha CD95 d.k.o. pool was generated by Synthego using the guide RNA sequence: GGAGUUGAUGUCAGUCACUU. Single cells were sorted into 96-well plates with 50% conditioned media by FACS. Colonies were treated with LzCD95L for 24 h, and visually inspected for signs of apoptosis. Resistant clones were expanded and assayed for DNA editing by PCR. The following PCR primers were used to confirm deletion, forward: tggtgctgtttctagtgTGGt, reverse: TGTTGCTACTCCTAACTGTGACT (design: Synthego, Synthesis: IDTDNA, custom DNA oligos with standard desalting). ChoiceTaq Blue (Denville cat #C775Y30) master mix was used for PCR amplification following manufacturer’s protocol. The QIAquick PCR Purification Kit (Qiagen cat. #28,106) was used to isolate the PCR product following the manufacturer’s protocol. The forward primer was submitted with the PCR product for Sanger sequencing (ACGT Inc.) The Synthego ICE CRISPR Analysis tool was used to analyze the editing efficiency (Synthego).

### CD95L lentiviral vectors

Generation of pLenti CD95L, pLenti CD95L NP, pLenti CD95L SIL, and pLenti CD95L Zero was described in [16]. All CD95L sequences were sub-cloned into the pLenti-GIII-CMV-RFP-2A-Puro vector (ABM Inc.). CD95L GUA was synthesized with NheI and XhoI flanking restriction enzyme sites and sub-cloned (GenScript). CD95L GUA contains the Y218R mutation (TAT > CGT) [1] and 13 in-frame Met > Val mutations (AUG > GUA).

### Lentivirus transduction experiments

Lentiviruses were packaged in 293T cells using psPAX2 (Addgene #12,260) and pMD2.G (Addgene #12,259) packaging plasmids. 293T cells were seeded to 90% confluency in 10 cm cell culture treated plates (Greiner Bio-ONE, cat #664,160). Plasmids were introduced by transfection with 60 µl Lipofectamine 2000 (ThermoFisher Scientific, #11,668,027) diluted in 2 ml Opti-MEM (Gibco cat, # 31,985–070) and 6 µg psPAX2, 3 µg pMD2.G packaging vectors, plus 10 µg delivery vectors, pLenti (ABM Inc.) or pTIP [12]. Media was refreshed 18 h later, and virus supernatant was collected after another 24 and 48 h. Viral supernatant was centrifuged and filtered through a 0.45 µm PVDF membrane (MilliporeSigma, SLHVM33RS) to remove debris, and stored at -80 °C. Mission shRNA Lentiviral particles designed to target CD95L were purchased: shL3 (Sigma TRCN0000059000), shL1 (Sigma TRCN0000058999), and shScr (Sigma SHC002V). All transductions were performed in the presence of 8 µg/ml polybrene. HCT116 cells were resuspended in media with polybrene with 150,000 cells seeded in 24-well plates, 300,000 in 12-well or 500,000 cells seeded in 6-well plates. Virus was added by volume with either 25% or 50% virus optimized by viral titer. For commercial viruses the volume required to achieve a multiplicity of infection (MOI) of 2–3 was added to each well. Cells were incubated at 37 °C, 5% CO_2_ overnight, and the viral media was replaced with regular media. Cells were treated with 2 µg/ml puromycin when RFP reporter expression was visible, usually 48–72 h post infection. Cells were selected for 48 h. After selection cells were reseeded for assays (time point 0).

### Transfection with short oligonucleotides

Custom siRNA oligonucleotides were ordered from integrated DNA technologies (IDT) as described previously: siCD95L cluster 15.1 and 15.2 [16], siL3 [11], siGGGGGC [13]. The non-targeting control siNT8 anti-sense 5’-UAAUCUAACAUGUAAACCAAA-3’, sense 5’- mUmGGUUUACAUGUUAGAUUATT-3’. A lowercase m before the base indicates 2’-O-methylated nucleotides. The oligos were annealed according to the manufacturer’s instructions. Duplexed siRNAs were introduced via reverse transfection using RNAiMax diluted in Opti-MEM in 96-well plates. A 50 μl transfection mix was added per well with a final siRNA concentration of 10 nM. The optimal volume of RNAiMax was determined for each clone, 0.2 or 0.3 μl was used. Diluted cells (3,000 – 8,000) were plated in 200 μl in each well on top of the transfection mix to a final volume of 250 μl. The effect on cell growth and viability was assayed as described below.

### Ago2 knockdown experiments

HCT116 wt and Drosha k.o. cells were reverse transfected with 25 nM siRNA SMARTpool targeting siAgo2 (Dharmacon, L-004639–00-0005) or a control siRNA pool (siCtr, Dharmacon, D-001810–10). The transfection mix was prepared in 500 μl Opti-MEM with 2 μl RNAiMax. Cells were seeded 500,000 cells in 1.5 ml media. The cells and transfection mix were combined in 6-well plates. Cells were incubated at 37 °C, 5% CO_2_ overnight, and the media was replaced the following day. 48 h post-transfection) cells were transduced with 25% pLenti of pLenti CD95L NP, cells were seeded in 12-well plates with 250,000 cells per well in 750 μl media, 8 μg/ml polybrene and 250 μl of virus was added. After an incubation at 37 °C, 5% CO_2_ overnight the viral media was replaced. Cells were treated with 2 μg/ml puromycin for 48 h and then plated for IncuCyte Zoom analysis of cell growth. HCT116 Drosha CD95 d.k.o. clone #12 were prepared with the following modifications. To knockdown Ago2, cells were reverse transfected in 24-well plates, 150,000 cells were plated per well. The SMARTpool siRNAs were diluted to 20–25 nM per well, and cells were transduced with 50% pLenti or pLenti CD95L NP viruses. For shRNA transductions, cells were infected with pLKO-shScr or shL3 at an MOI of 3.0. Uninfected cells were harvested 96 h after transfection to assess the efficiency of Ago2 knockdown. Cell viability was determined 120 h after puromycin selection. MCF7 CD95 k.o. cells were reverse transfected in 12-well plates with 150,000 cells seeded per well. RNAiMax, 3 μl per well, and SMARTpool siRNAs (25 nM) were diluted in 250 μl Opti-MEM. Cells were infected with 50% pLenti or pLenti CD95L NP virus and centrifuged at 2700 rpm for 20 min. Cells were treated with puromycin 48 h later and selected for 72 h before plating for IncuCyte and ATP assay (assessed at 96 h).

### Analysis of cell growth and viability

Cells were seeded in 96-well plates, 2000 to 8000 cells per well, in triplicates or quadruplicates. For analysis by IncuCyte cells were seeded in TPP tissue culture test plates (TPP, cat #92,696). For analyses of ATP content cells were plated in either white CELLSTAR tissue culture microplates (Greiner, BIO-ONE, cat # 655,083) or μCLEAR Black, CELLSTAR, tissue culture microplates (Greiner, BIO-ONE, cat # 655,090). Cell growth over time was analyzed using the IncuCyte ZOOM live-cell imaging system (Essen BioScience). Images were acquired every 4–6 h using a 10 × objective. The IncuCyte ZOOM software (version 2015A) was used to process images. Growth was analyzed as a change in cell confluency over time.

Cell viability was determined by CellTiter-Glo 2.0 Cell Viability Assay according to the manufacturer’s instructions (Promega, cat # G9241). Unless otherwise indicated, ATP content was assayed at 96 h after seeding. Briefly, media was removed to reduce the cell culture media to 75 μl per well and 75 μl of the CellTiter-Glo reagent was added. The plate was agitated on an orbital shaker for 2 min and luminescence was read after a 15-min incubation at room temperature using the BioTek Synergy Neo2.

### Real-time PCR

To make cDNA, the High-Capacity cDNA Reverse Transcription Kit (Applied Biosystems #4,368,814) was used with 200 ng input total RNA, determined by nanodrop readings (Nanodrop 2000). The qPCR reaction was performed using Taqman gene expression mix (ThermoFisher Scientific, #4,369,016) and the following FAM Taqman probes: GAPDH (Hs00266705_g1), ACTB (Hs01060665_g1), human CD95L (Hs00181226_g1 and Hs00181225_m1), and a custom probe to detect the CD95L SIL (Thermofisher Scientific, assay ID: APNKTUD [16]). Reactions were performed in triplicate in 96-well plates (Applied Biosystems, cat # N8010560) in the Applied Biosystems 7500 Real Time PCR system. CT values were determined using default settings, and relative expression was determined using the ΔΔCT method normalized to samples expressing pLenti CD95L wild type.

### Western blotting

To confirm Ago2 knockdown, cells were lysed in Killer RIPA lysis buffer (150 mM NaCl, 10 mM Tris–HCl pH 2.7, 1% SDS, 1% Triton X-100, 1% deoxycholate, 5 mM EDTA) with freshly added protease inhibitor cocktail (Roche, cat #11,836,170,001). Lysates were passed through a 26G syringe and protein was quantified by DC Protein Assay (Bio-Rad, cat #5,000,112). The lysates were then diluted to equal volumes and 30 μg protein was loaded per well using the Novex WedgeWell Tris–Glycine Welcome Pack (ThermoFisher Scientific, cat #XP0420B). The SDS-PAGE gel was transferred to a nitrocellulose membrane (Sigma, cat #GE10600016). Blots were blocked in 5% milk TBS-T (0.1% Tween-20/TBS) and incubated overnight at 4 °C in primary antibodies diluted in 5% milk TBS-T: anti-Ago2 (1:500–1000), anti-Ago4 (1:1000), anti-Actin (1:5000). The blots were incubated in the secondary antibody for 2 h at room temperature, anti-rabbit IgG HRP diluted (1:5000) in 5% milk TBS-T. Protein bands were visualized using the SuperSignal West Dura Extended Duration Substrate (ThermoFisher Scientific, cat #34,076) captured by the G:Box Chemi XT^4^ imager (Syngene).

### CD95 surface staining

Cells were resuspended in 300 μl 5% BSA in PBS with 5 μl of anti-CD95 or IgG control. Cells were incubated for ~ 4 h on ice. Then cells were fixed in 4% formaldehyde in PBS for 20 min. Cells were washed three times with PBS and resuspended in 5% BSA in PBS. Cells were kept on ice and analyzed on the BD LSRFortessa SORP Cell Analyzer with HTS.

### Sample preparation for Ago-RP-Seq

Spin infections were performed with cells in suspension in 6-well plates. HCT116 cells (wt, Drosha k.o., Dicer k.o. and Drosha CD95 d.k.o. clone #12) were passed through a 40 μm cell strainer and diluted to 500,000 cells per ml in media with 16 μg/ml polybrene. Cells were plated with 1 ml virus or DMEM media control (final concentration of polybrene 8 μg/ml) in 6-well plates with 20–36 wells infected per virus. The plates were centrifuged at 2700 rpm for 20 min at room temperature, and incubated at 37 °C, 5% CO_2_ overnight. The next day cells were trypsinized and pooled, 10–18 wells per sample replicate (two sample replicates per virus) and replated in 15 cm dishes. Cells were treated with 2 μg/ml puromycin 48–72 h after infection. Cells were expanded as needed. After 48 h of puromycin selection cells were plated for IncuCyte analysis and ATP assay. On day 5 (~ 122 h post-infection), cell pellets were harvested. Cells were trypsinized, pelleted, washed with PBS and counted. Aliquots of 8–10 million cells were pelleted and flash frozen for Ago-RP.

### Ago-RP and library preparation for small RNA-Seq

Pulldown of Ago 1–4 was performed as previously described [16]. Briefly, cell pellets were lysed in 1 ml NP-40 lysis buffer (50 mM Tris pH 7.5, 150 mM NaCl, 5 mM EDTA, 0.5% NP-40, 10% (v/v) glycerol, 1 mM NaF; supplemented with 1:200 EDTA-free protease inhibitors (Millipore, #539,134) and 1:1000 RNaisin Plus (Promega, #N2615). Samples were vortexed and kept on ice ~ 30 min. Cell debris was removed by centrifugation at 20,000 × G for 20 min. at 4 °C. Lysates were transferred to Lo-bind tubes (Eppendorf, #022,431,021) and incubated with 500 µg Flag-GST-T6B peptide [49] and 80 μl of anti-Flag M2 Magnetic beads (Sigma, #M8823) for at least 3 h rotating at 4 °C. Beads were washed three times with 1 ml NP-40 lysis buffer. After the last wash, 100 μl were aliquoted for western blot analysis to confirm the efficiency of Argonaute pulldown. The NP-40 lysis buffer was removed and beads were resuspended in 500 μl Trizol (Ambion, #15,596,018). RNA was extracted according to the manufacturer’s protocol, resuspended in 20 μl UltraPure water and divided into two 10 μl aliquots.

Size markers 19 and 35 nucleotides long were dephosphorylated using 0.5 U/ μl CIP alkaline phosphatase (NEB M0290L) by incubating at 37 °C for 15 min, and then radiolabeled with 0.5 μCi [γ-^32^P]ATP (Perkin Elmer NEG002Z250UC) using T4 PNK kinase (NEB M0201L) incubating at 37 °C for 20 min. The size markers were resolved on a 15% urea-PAGE gel (National Diagnostics, EC830, EC835, EC840), extracted, resuspended in UltraPure water for use in subsequent steps. The RNA isolated from the Ago-RP was then prepared for small RNA sequencing as previously described [50]. Briefly, the RNA and the size marker was ligated with Barcoded 3’ adenylated adapters using the T4 RNA Ligase 2, truncated K227Q (NEB, #M0351) for at least 3 h at 16 °C. The ligation products were pooled into one tube and isolated by ethanol precipitation. The RNA-adapter pellet was dissolved in 20 μl water and resolved on a 15% urea-PAGE gel flanked by radiolabeled size markers with adapters. RNA the size of the shifted size markers was excised and a gel extraction was performed. The RNA-adapter pellet was resuspended in 9 μl UltraPure water. A 5’ adapter was ligated using T4 RNA ligase, T4 Rnl1 (Thermofisher, cat #EL0021) incubated at 37 °C for 1 h while agitating. The product was resolved on a 12% urea-PAGE gel flanked by 3’ and 5’ ligated radiolabeled size markers. The small RNA adapter product was excised and a gel extraction was performed. The RNA-adapter pellet was resuspended in 4.6 μl UltraPure water, and reverse transcribed using the Superscript III reverse transcriptase (Invitrogen, cat #18,080–044). The cDNA was amplified by PCR using Platinum™ *Taq* DNA Polymerase (Invitrogen, cat #10,966–018). Single end sequencing was performed on the Illumina Hi-Seq 4000.

### Processing of small RNA-Seq data

The raw small RNA-seq data was processed as previously described [16]. Illumina adapter sequences were removed with trim_galore. Unique Molecular Identifiers (UMIs) four nucleotides 5’ of the sequenced small RNA and two nucleotides 3’ of the sequence were removed. Tophat was used to align reads to the hg38 assembly of the human genome. Raw read counts were assigned to genes using HTseq. Gene expression was normalized based on library size and sequence complexity using EdgeR.

### Identification of CD95L reads

CD95L reads were identified as previously described [16]. Briefly, the reads from each sample were compiled as a BLAST database, and blastn was utilized to match the CD95L ORF (wild type NM_00639.2 or CD95L mutant sequences) to reads in each sample. Reads were considered matches if they had an e-value < 0.05% and 100% identity across the length of the read. These hits were converted to bed files that were used for subsequent analyses. Sample replicates were combined and stack plots depicting the locations where RISC bound CD95L sRNAs map along the CD95L ORF were generated using the R package Sushi.

### Analysis of CD95L read abundance

CD95L read abundance was determined by tallying raw matching reads identified using blastn. CD95L reads were counted for each sample from individual replicate bed files. The total number of raw reads per sample was calculated, as was the percent contribution of CD95L reads to the total read counts.

### Identification of shL3 reads

The presence of CD95L-derived sRNAs with the same sequence as commercial CD95L targeting shRNAs was queried by searching the bed files for reads that contain the 5-p arm of the shRNA. Reads mapping to cluster 15 in the CD95L ORF contained the shL3-5p sequence (with the same ‘5 start site, -3, + 1) were tallied and were highlighted in orange in Fig. 1A using Sushi. The percentage of reads mapping to shL3 from CD95L was calculated, and the variation in the 5’ end of mapping reads was depicted in the pie chart in Fig. 1B.

### CD95L mRNA processing analysis

Analysis of small RNA-Seq data of shL3 infected cells was described previously [11]. CD95L read length was determined from sample bed files and tallied using the tidyverse package in R. For the analysis of the start and stop site of abundant stacks in HCT116 wt, Drosha k.o., and Dicer k.o. cells the top 10 most abundant stacks were identified. The most abundant read was annotated as starting at position 0 based on the mapping of the 5’ end of the read. Using the mapping of the 5’ start site indicated in sample bed files the number of reads mapping -5 to + 5 nucleotides was tallied, and plotted as line plots using ggplot2 in R. To analyze processing at the 3’ end of each read, reads starting at position 0 were analyzed. The read length of each was tallied and also plotted as line plots.

### CD95L stem-loop and nucleotide analysis

RNAfold from the ViennaRNA package was used to predict the secondary structure of CD95L mRNA and the various CD95L mutants [51]. The RNAfold Dot-Bracket output was imported into R. A barplot of height equal to 1 (y-axis) was generated using the package ggplot2 with one bar representing each nucleotide in the CD95L ORF (846 in total on the x-axis). The color of the bars corresponds to the Dot-Bracket notation, blue indicates unpaired nucleotides (dots), and red bars indicate paired nucleotides (brackets). The same strategy was employed to visualize the nucleotide composition of the CD95L sequences. Again, ggplot2 was used to generate a bar plot with an x-axis 1–846 and a y-axis equal to 1. The color of the bars represent different nucleotides bases in the CD95L sequence with Adenines in blue, Cytosines in yellow, Guanines in red, and Uracils in green.

### CD95L 6mer seed toxicity analysis

The six nucleotide seed sequence (positions 2–7) was extracted from the reads in the CD95L bed files. The 6mer seed viability data corresponding to each 6mer seed sequence from the average of three human cell lines (6mer db.org) was added in R using the tidyverse package. The viability data was summarized in boxplots using ggplot2. Only reads 18–25 nucleotides long were included in the analysis. A breakdown of the contribution of different seed sequences to the overall seed viability was profiled by binning the viability data and counting the number of reads expected to exert the same effect on cell viability. The viability data was rounded to the nearest integer, and the number of reads with the same viability was tallied. The binned data was then plotted in Excel with viability on the x-axis and read counts on the Y-axis as described in [14].

### Identification of processed mRNAs

For each sample, small RNA-Seq reads were aligned to the human genome build hg38 using Tophat and converted to.bam files. Using blastn, each bam file was compiled into a blast database. A fasta file containing the longest annotated transcript of each protein coding gene was queried against each sample database. Matches were filtered for 100% identity and an e-value < 0.05%. Only uniquely mapping reads were considered hits. Regions with greater than 10 reads mapping with the same 5’ start site were defined as a stack. The number of stacks, or the stack count, across a gene transcript was tallied, and the stack counts were merged with the normalized RNA-Seq tables. The table was annotated with gene biotypes information from Ensembl using biomart. A processed mRNA was identified as any gene annotated as protein-coding with three or more stacks and 10 or more normalized reads in averaged sample replicates. Scatterplots representing stacks by normalized gene expression were generated in R using ggplot2. Dark blue data points represent genes that were common in HCT116 wt, Drosha k.o. and Dicer k.o. cells. A Venn diagram was generated using the web-based application BioVenn [52]. The Database for Annotation, Visualization and integrated Discovery (DAVID) release v2022q2 was utilized to analyze the processed gene list from each genotype for enriched gene ontologies [53, 54]. Gene ontologies were annotated using the GOTERM_BP_FAT. Common GOterms with greater than 1.5 fold enrichment and a Bonferroni adjusted *p*-value < 0.05 were considered to be significantly enriched. The top 10 processed genes described in Fig. S8 were identified by ranking processed mRNAs by stack count and expression. Genes ranked in the top 50 in both the Ago pulldown and total small RNA-Seq data were selected for further curation. Genes with reads annotated in miRBase (v. 22.1) as miRNAs were excluded. Also, genes with reads mapping primarily to one location were excluded.

### Analysis of reads from processed mRNAs

As described for CD95L, BLAST databases compiled for each sample replicate were queried for matches to select protein-coding mRNA transcripts. Hits with an e-value < 0.05% and 100% identity across the read were compiled in bed files. Bed files were concatenated and plotted using the Sushi package in R. For analysis of the predicted effect of mRNA reads on cell fate, the six nucleotide seed sequence (positions 2–7) was extracted from each read and the 6mer seed viability data, averaged across human samples, corresponding to each seed sequence was matched. A dataframe was generated with the 6mer seed viability “score” printed one time for each read in a sample. The data was represented as density plots, boxplots or violin plots using the R package ggplot2.

### Statistical analyses

Student’s T-tests were performed in Microsoft Excel. Two-way analyses of variance were evaluated using STATA version 14.0. STATA was also used for comparing differences in polynomial distributions as described previously [10]. All other statistical analyses were conducted in R version 4.0.0.

## Supplementary Information

**Additional file 1:** **Supplementary Fig. 1.** Processing of pTIP-shL3 results in preferential selection of the sense strand of the shRNA.

**Additional file 2:** **Supplementary Fig. 2.** Dicer is not required for pLenti CD95L NP toxicity.

**Additional file 3:** **Supplementary Fig. 3.** Characterization of R-sRNAs in cells lacking Dicer and Drosha.

**Additional file 4:** **Supplementary Fig. 4.** Endogenous mRNAs are processed and loaded into the RISC of Dicer k.o. cells.

**Additional file 5:** **Supplementary Fig. 5.** Characterization of HCT116 Drosha CD95 d.k.o. cells.

**Additional file 6:** **Supplementary Fig. 6.** The role of Ago2 in mediating CD95L toxicity is cell type specific.

**Additional file 7:** **Supplementary Fig. 7.** Reanalysis of the qPCR data in Fig. 5b and 6b normalized to ACTB.

**Additional file 8:** **Supplementary Fig. 8.** CD95L-derived reads skew more toxic than reads derived from other mRNAs.

**Additional file 9:** **Supplementary Fig. 9.** Processing and RISC loading of CD95L mutant derived reads.

**Additional file 10:** **Supplementary Fig. 10.** Uncropped Western blots of Figure 4a and 5c.

## Data Availability

The data of this study are available from the corresponding author on reasonable request. Ago-RP-Seq datasets are publicly available, GSE219226. The following datasets were reanalyzed: GSE87817 and GSE114425 in Fig. 1 and Supplementary Fig. 1, respectively.

## Abbreviations

3'UTR: 3' Untranslated region
Ago: Argonaute
Ago-RP-Seq: Ago RNA precipitation combined with small RNAseq
DISE: Death induced by survival gene elimination
k.o.: Knock-out
NMD: Nonsense mediated decay
RISC: RNA induced silencing complex
RNAi: RNA interference
sRNA: Short RNA
R-sRNA: RISC bound sRNA
siRNA: Small interfering RNA
miRNA: MicroRNA
shRNA: Short interfering RNA
wt: Wild-type
