# Supplementary material for: CD95/Fas ligand mRNA is toxic to cells through more than one mechanism
Source: Mol Biomed. 2023 Apr 15;4:11. doi: 10.1186/s43556-023-00119-1 (PMC10105004; doi:10.1186/s43556-023-00119-1)
Supplement: Supplementary file 1 — Additional file 1: Supplementary Fig. 1. Processing of pTIP-shL3 results in preferential selection of the sense strand of the shRNA. [file 43556_2023_119_MOESM1_ESM.pdf]

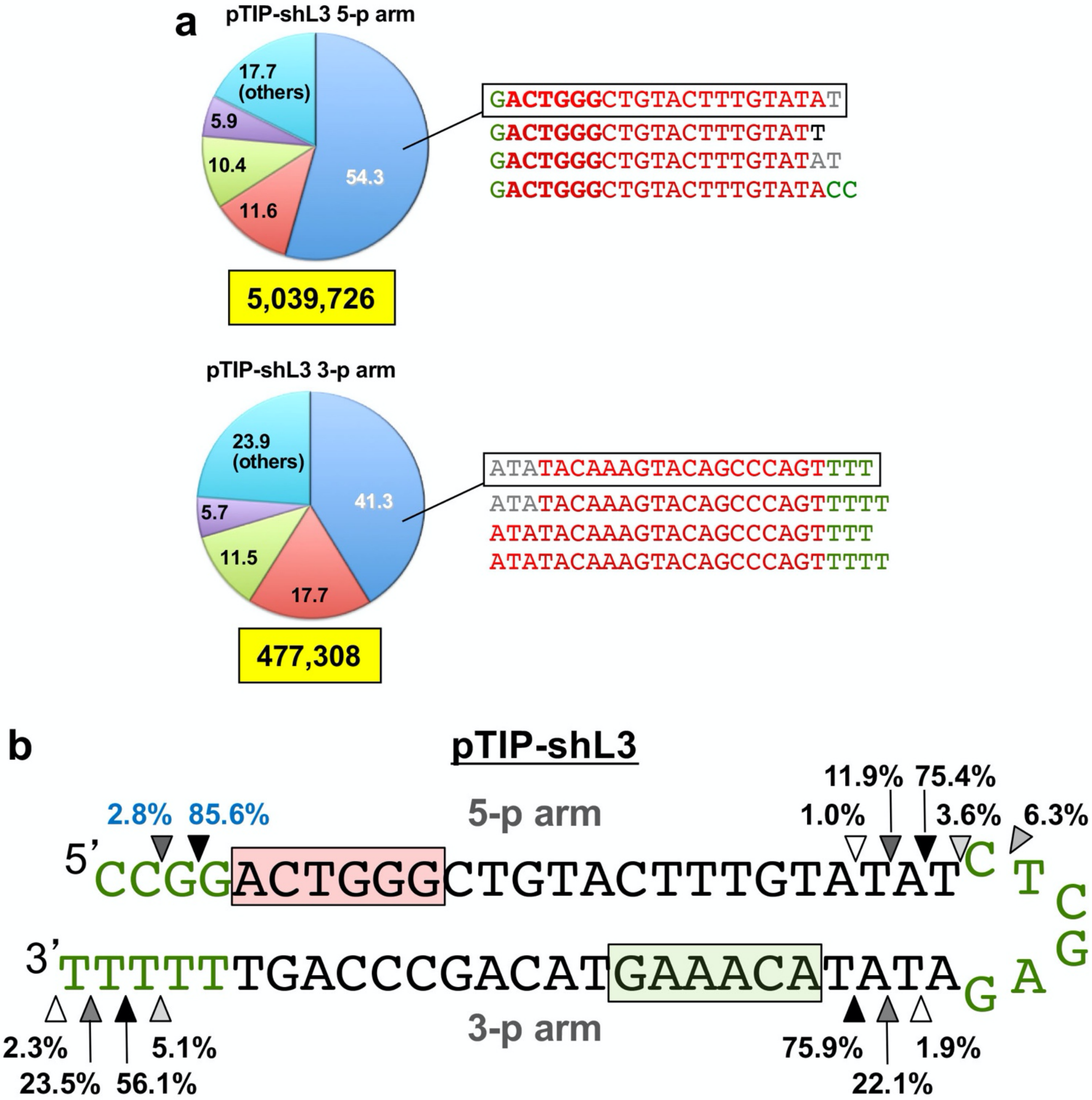

Figure S1 - Processing of pTIP-shL3 results in preferential selection of the sense strand of the shRNA
Reads mapping to shL3 in 293T stably expressing pTIP-shL3 50 hours after dox induction. **(a)** Pie charts representing the contribution of each shL3-derived sequence to the total reads derived from the two shRNA arms. Sequences derived from the pTIP-shL3 5-p arm (sense strand, top) and the 3-p arm (antisense strand, bottom) are shown. The top four most abundant sequences derived from each arm are listed. Nucleotides shown in green are derived from the viral vector, in red are shRNA sequences, in grey are missing nucleotides, and in black are additional nucleotides. **(b)** Schematic representing the secondary structure of shL3. The frequency of each cleavage event in the RNA-Seq data and the site of cleavage is indicated with arrows. The most common cleavage site is indicated with a black arrow, and the least common with a white arrow. Nucleotides derived from the pTIP shRNA vector are in green. The 6mer seed of the most abundant generated shRNA on the 5-p arm is indicated in the red box, the one on the 3-p arm in a green box.
