# Supplementary material for: CD95/Fas ligand mRNA is toxic to cells through more than one mechanism
Source: Mol Biomed. 2023 Apr 15;4:11. doi: 10.1186/s43556-023-00119-1 (PMC10105004; doi:10.1186/s43556-023-00119-1)
Supplement: Supplementary file 2 — Additional file 2: Supplementary Fig. 2. Dicer is not required for pLenti CD95L NP toxicity. [file 43556_2023_119_MOESM2_ESM.pdf]

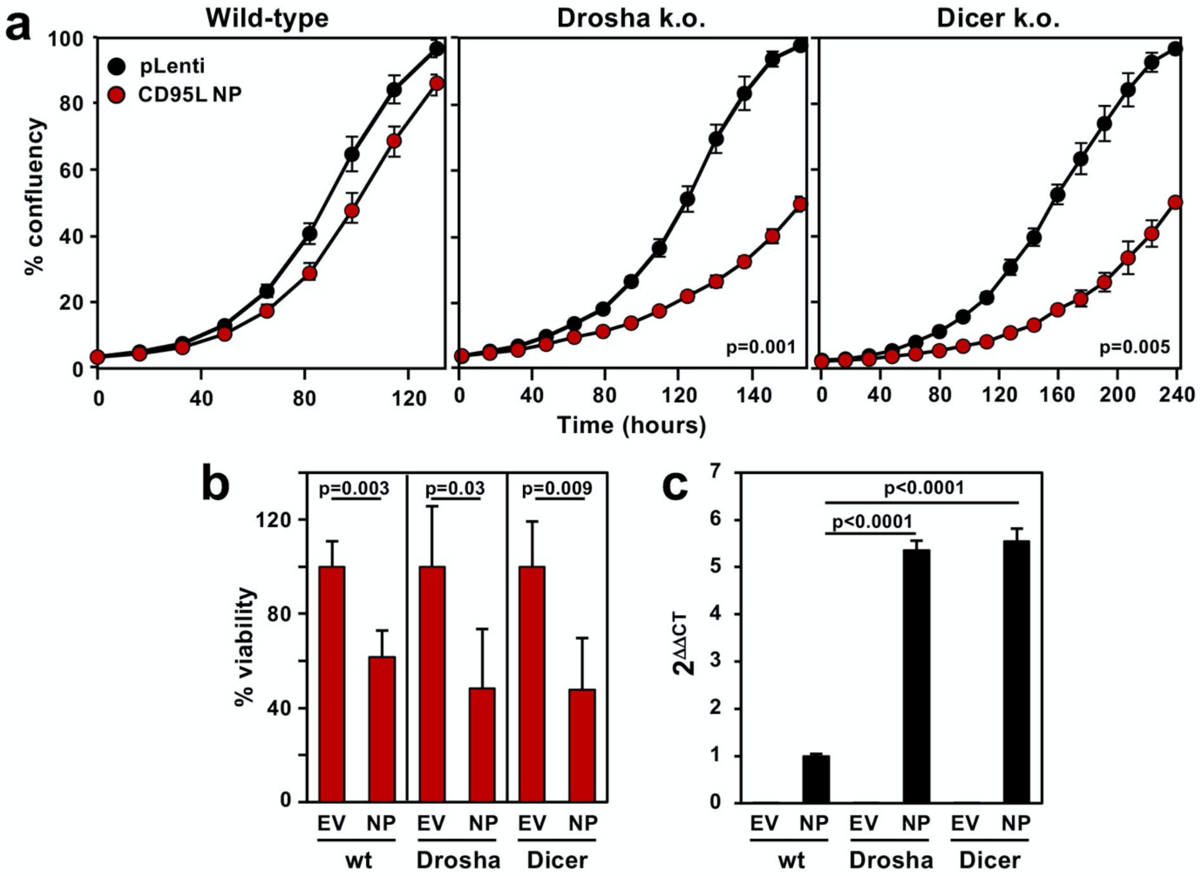

Figure S2 - Dicer is not required for pLenti CD95L NP toxicity
(a) Percent confluency over time of HCT116 wt (left) HCT116 Drosha k.o. (center) and HCT116 Dicer k.o. cells (right) expressing either pLenti or pLenti-CD95L NP. Data are representative of >3 independent experiments. P-values were calculated using a polynomial distribution test and support a difference in sensitivity between wild-type HCT116, Dicer and Drosha k.o. cells. Each data point represents mean ± SE of three replicates. **(b)** Percent viability of the cells 120 hrs after infection. Viability of CD95L expressing cells was normalized to pLenti (EV) expressing cells of the same genotype. Error bars represent the standard deviation (SD) of three replicates. T-test, Benjamini corrected p-value. **(c)** Real-time qPCR assessment of CD95L mRNA expression in wild-type cells of each genotype. Expression was normalized to HCT116 cells expressing pLenti-CD95L NP and GAPDH. Error bars represent the SD of sample triplicates. Data representative of >3 independent experiments. T-test, Bonferroni adjusted p-value.
