# Supplementary material for: CD95/Fas ligand mRNA is toxic to cells through more than one mechanism
Source: Mol Biomed. 2023 Apr 15;4:11. doi: 10.1186/s43556-023-00119-1 (PMC10105004; doi:10.1186/s43556-023-00119-1)
Supplement: Supplementary file 3 — Additional file 3: Supplementary Fig. 3. Characterization of R-sRNAs in cells lacking Dicer and Drosha. [file 43556_2023_119_MOESM3_ESM.pdf]

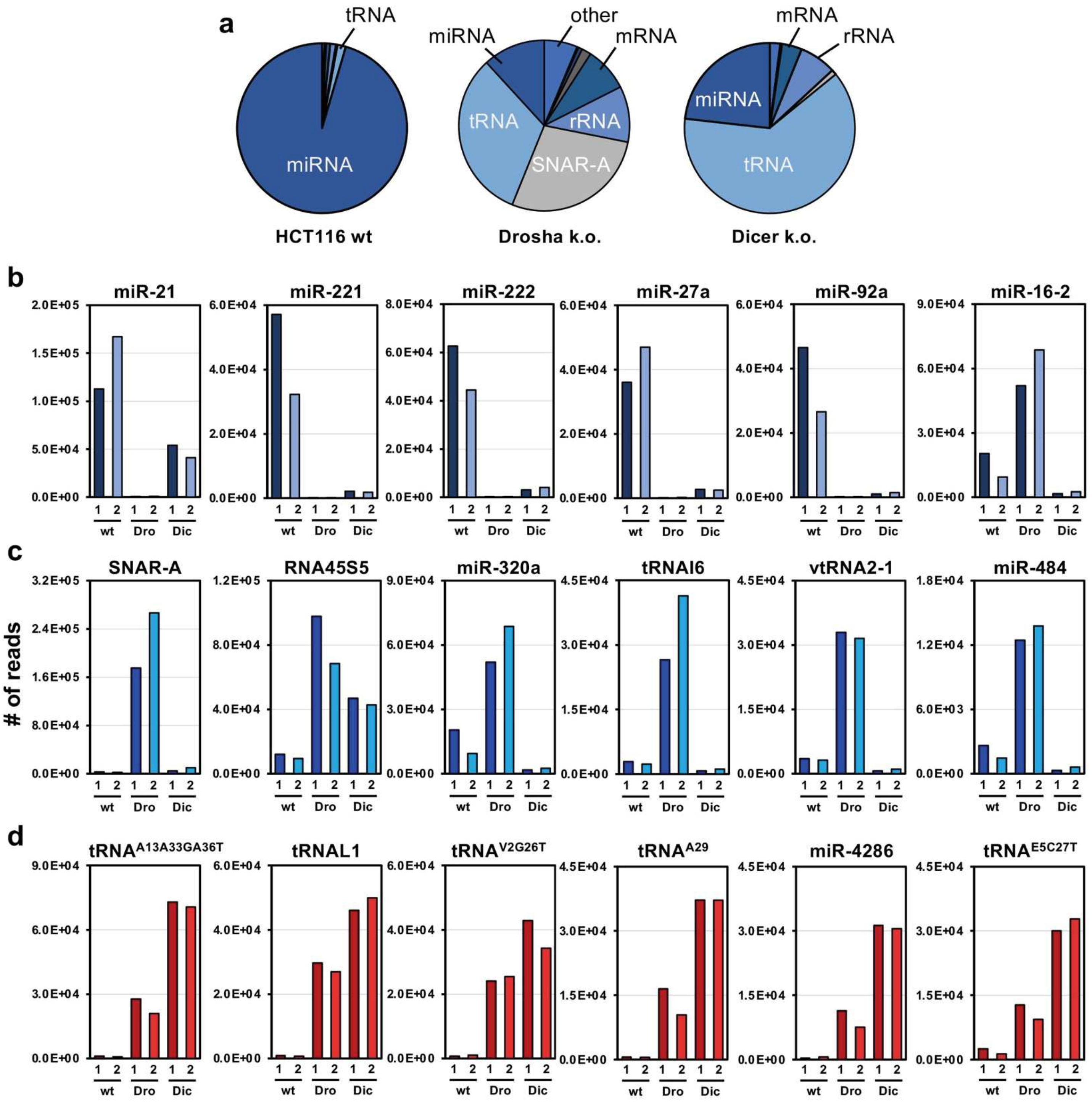


**Figure S3 - Characterization of R-sRNAs in cells lacking Dicer and Drosha**
**(a)** Pie charts representing the types of sRNAs that occupy the RISC of HCT116 cells by genotype. **(b)** Number of reads normalized per million of the top five most abundant sRNAs in the RISC of HCT116 cells (navy), **(c)** HCT116 Drosha k.o. cells (blue), and **(d)** HCT116 Dicer k.o. cells (red). Each bar represents read counts in one sample replicate, sample replicate 1 (darker shade) and replicate 2 (lighter shade).
