# Supplementary material for: CD95/Fas ligand mRNA is toxic to cells through more than one mechanism
Source: Mol Biomed. 2023 Apr 15;4:11. doi: 10.1186/s43556-023-00119-1 (PMC10105004; doi:10.1186/s43556-023-00119-1)
Supplement: Supplementary file 4 — Additional file 4: Supplementary Fig. 4. Endogenous mRNAs are processed and loaded into the RISC of Dicer k.o. cells. [file 43556_2023_119_MOESM4_ESM.pdf]

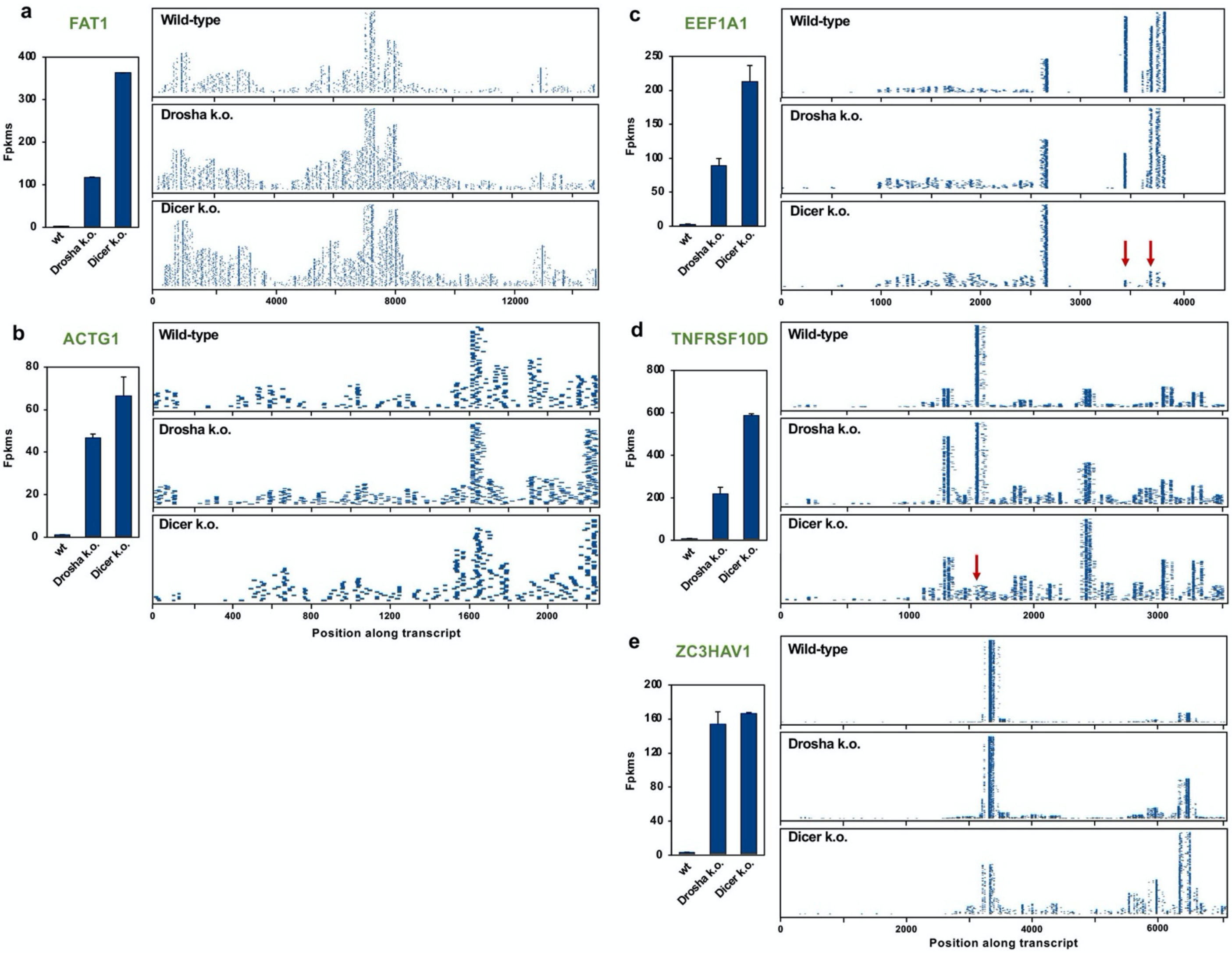

Figure S4 - Endogenous mRNAs are processed and loaded into the RISC of Dicer k.o. cells
(a-e) Mapping of R-sRNAs to the transcripts of five selected highly processed mRNAs in cells infected with pLenti-CD95L NP. One horizontal line represents one read in the RISC of HCT116 wild-type (top), Drosha k.o. (center), and Dicer k.o. cells (bottom). *Left*, Reads from both replicates are combined and error bars show SD. *Right*, normalized read counts (fpkms) by genotype. Selected processed mRNAs **(a)** FAT1, **(b),** ACTG1, **(c)** EEF1A1, **(d)** TNFRSF10D, and **(e)** ZC3HAV1. Red arrows indicate stacks with few reads in Dicer k.o. cells.
