# Supplementary material for: CD95/Fas ligand mRNA is toxic to cells through more than one mechanism
Source: Mol Biomed. 2023 Apr 15;4:11. doi: 10.1186/s43556-023-00119-1 (PMC10105004; doi:10.1186/s43556-023-00119-1)
Supplement: Supplementary file 5 — Additional file 5: Supplementary Fig. 5. Characterization of HCT116 Drosha CD95 d.k.o. cells. [file 43556_2023_119_MOESM5_ESM.pdf]

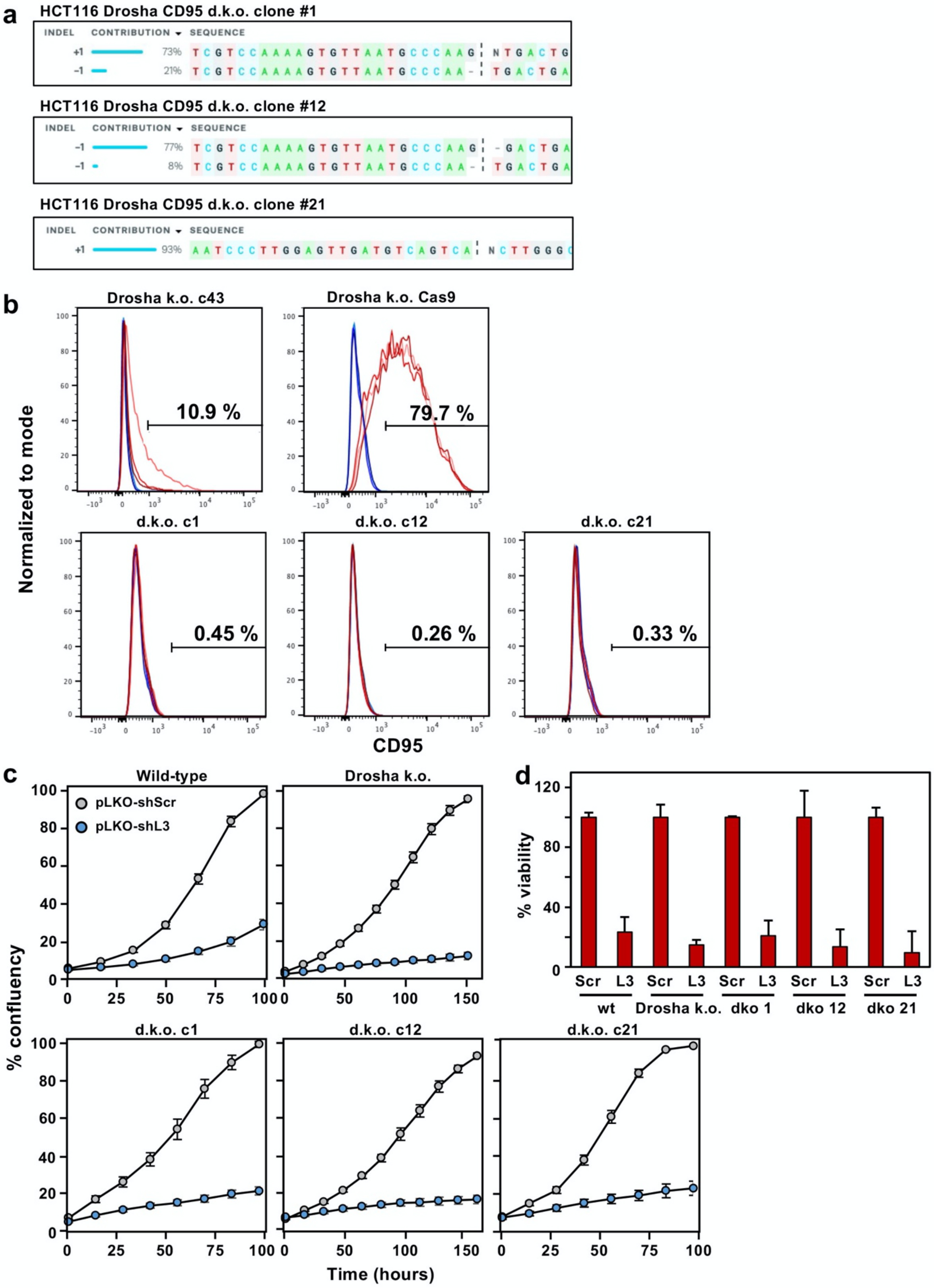

Figure S5 - Characterization of HCT116 Drosha CD95 d.k.o. cells

(a) Representation and contribution of indel mutations in the DNA of single cell clones analyzed by Synthego ICE tool. **(b)** Surface staining of HCT116 and Drosha CD95 d.k.o. clones for CD95. Anti-CD95 sample replicates (red) and IgG isotype controls (blue). Gates represent % positivity. (**c**) Cell confluency over time in HCT116 cells expressing either pLKO-shL3 or pLKO-shScr. HCT116 wild-type, Drosha k.o., and d.k.o. cells were assayed. Bars indicate standard error of triplicates. **(d)** Relative cell viability at 96 hours. Error bars represent standard deviation of triplicates.
