# Supplementary material for: CD95/Fas ligand mRNA is toxic to cells through more than one mechanism
Source: Mol Biomed. 2023 Apr 15;4:11. doi: 10.1186/s43556-023-00119-1 (PMC10105004; doi:10.1186/s43556-023-00119-1)
Supplement: Supplementary file 6 — Additional file 6: Supplementary Fig. 6. The role of Ago2 in mediating CD95L toxicity is cell type specific. [file 43556_2023_119_MOESM6_ESM.pdf]

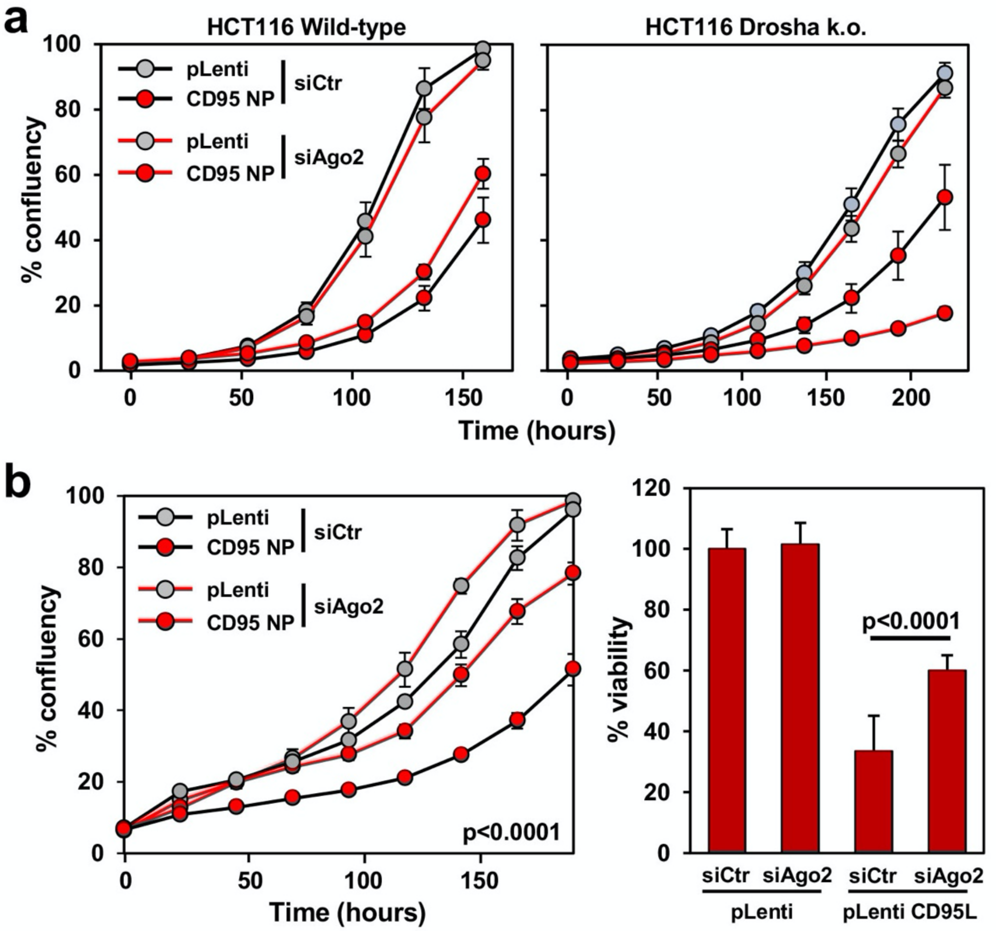

Figure S6 - The role of Ago2 in mediating CD95L toxicity is cell type specific
(a) Percent cell confluency over time in HCT116 wild-type (left) and HCT116 Drosha k.o. (right) cells transfected with 25 nM siAgo2 or siCtr and subsequently infected with pLenti or pLenti-CD95L NP. Data is representative of two independent experiments. **(b)** *Left*, Percent cell confluency over time in MCF7 CD95 k.o. cells transfected with 25 nM siNT or siAgo2 siRNAs and subsequently infected with pLenti or pLenti-CD95L NP. P-value represents Two-way ANOVA between CD95 NP sCtr versus siAgo2. *Right*, Relative viability of cells at 96 hours normalized to siCtr transfected cells infected with pLenti empty vector. Error bars represent standard deviation. Student's T-test is shown.
