# Supplementary material for: CD95/Fas ligand mRNA is toxic to cells through more than one mechanism
Source: Mol Biomed. 2023 Apr 15;4:11. doi: 10.1186/s43556-023-00119-1 (PMC10105004; doi:10.1186/s43556-023-00119-1)
Supplement: Supplementary file 7 — Additional file 7: Supplementary Fig. 7. Reanalysis of the qPCR data in Fig. 5b and 6b normalized to ACTB. [file 43556_2023_119_MOESM7_ESM.pdf]

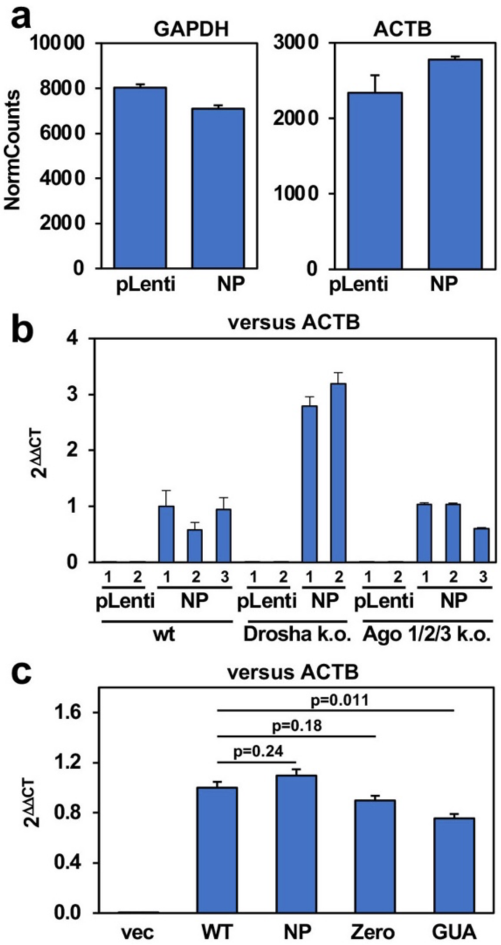

Figure S7 - Reanalysis of the qPCR data in Fig. 5b and 6b normalized to ACTB
(a) RNA-Seq analysis of GAPDH and ACTB expression in CD95 k.o. HeyA8 cells 50 hrs after infection with either pLenti or pLenti-CD95L (previous data deposited at GSE103631 were analyzed). Shown are the means of duplicate samples with SD. Raw read counts were normalized to 1 million reads per sample (NormCounts). **(b)** Reanalysis of the data shown in Fig. 5b normalized to ACTB, Bars represent the mean with standard deviation of triplicates. **(c)** Reanalysis of the data shown in Fig. 6b normalized to ACTB, Bars represent the mean with standard deviation of triplicates. T-test, Bonferroni adjusted p-value.
