# Supplementary material for: CD95/Fas ligand mRNA is toxic to cells through more than one mechanism
Source: Mol Biomed. 2023 Apr 15;4:11. doi: 10.1186/s43556-023-00119-1 (PMC10105004; doi:10.1186/s43556-023-00119-1)
Supplement: Supplementary file 8 — Additional file 8: Supplementary Fig. 8. CD95L-derived reads skew more toxic than reads derived from other mRNAs. [file 43556_2023_119_MOESM8_ESM.pdf]

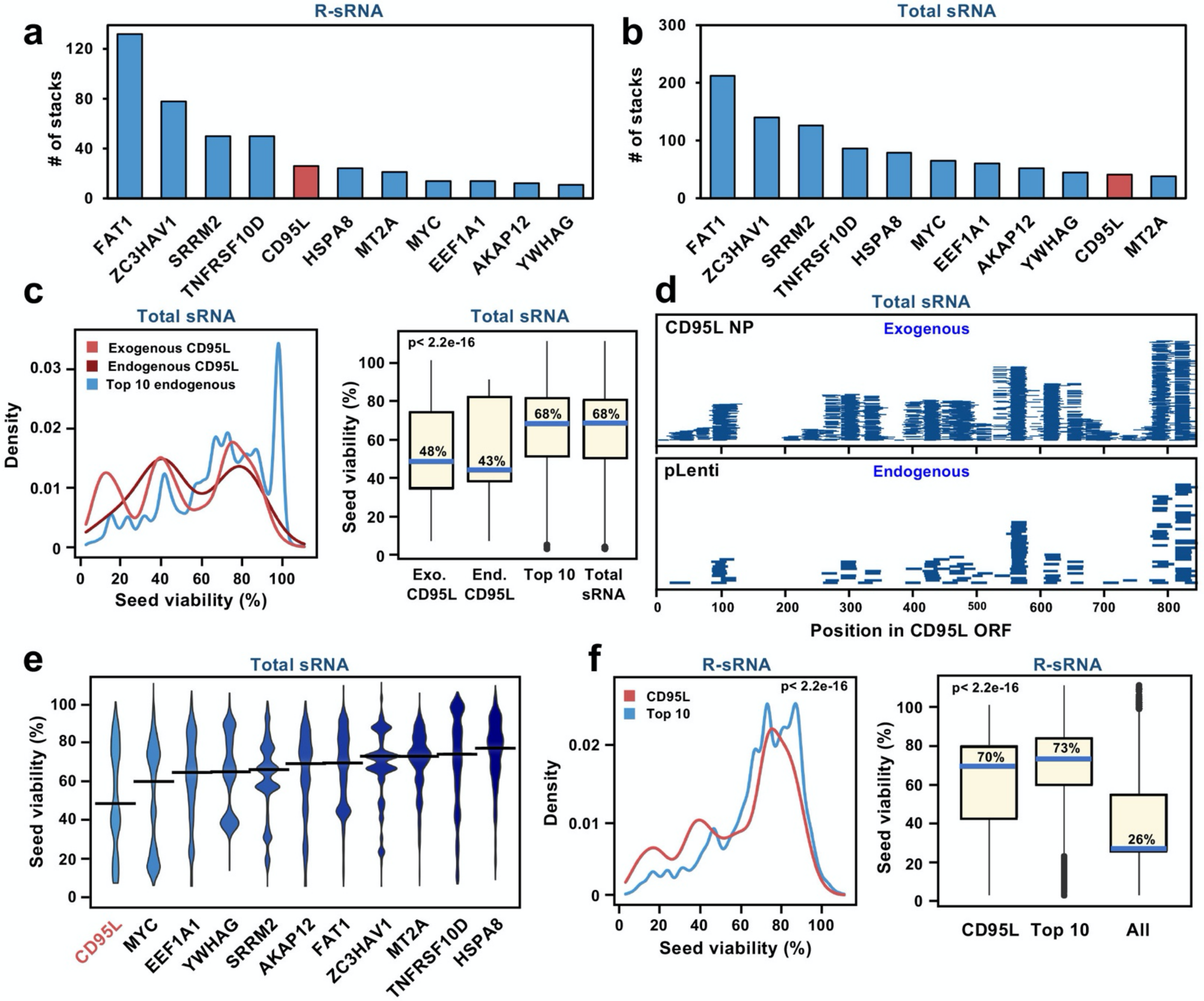

Figure S8 - CD95L-derived reads skew more toxic than reads derived from other mRNAs
(a, b) Ranking of highly expressed and processed protein coding genes by number of stacks. A stack was defined as reads, 10 or more, with the same 5' start site. pLenti-CD95L NP is indicated in red. Ago bound reads are represented in **(a)** and stacks in the total sRNA are represented in (b)**.** Reads from two replicates were combined. **(c)** *Left*, Density plot representing the predicted 6mer seed viability of reads 18-25 nt long derived from CD95L vs the Top 10 most abundant and highly processed mRNAs in the total sRNA in aggregate. *Right*, box plots representing the skew of the 6mer seed viability of the derived reads. Blue lines indicate the median 6mer seed viability. Kruskal-Wallis p-value is shown. **(d)** Mapping of CD95L-derived sRNAs found in the total sRNA to the CD95L ORF. Reads derived from exogenously expressed CD95L (top), and endogenous CD95L-derived reads (bottom). **(e)** Violin plots representing the distribution and the median (black solid line) 6mer seed viability of each of the Top 10 processed mRNAs and exogenously expressed CD95L (red). **(f)** *Left*, Density plot representing the predicted 6mer seed viability of R-sRNAs derived from CD95L vs the Top 10 processed mRNAs. Endogenous CD95L reads were too few to plot. *Right*, box plots representing the skew of the 6mer seed viability of R-sRNAs derived from CD95L, the Top 10 processed mRNAs and the total RISC content (Kruskal-Wallis p-values are shown).
