# Supplementary material for: CD95/Fas ligand mRNA is toxic to cells through more than one mechanism
Source: Mol Biomed. 2023 Apr 15;4:11. doi: 10.1186/s43556-023-00119-1 (PMC10105004; doi:10.1186/s43556-023-00119-1)
Supplement: Supplementary file 9 — Additional file 9: Supplementary Fig. 9. Processing and RISC loading of CD95L mutant derived reads. [file 43556_2023_119_MOESM9_ESM.pdf]

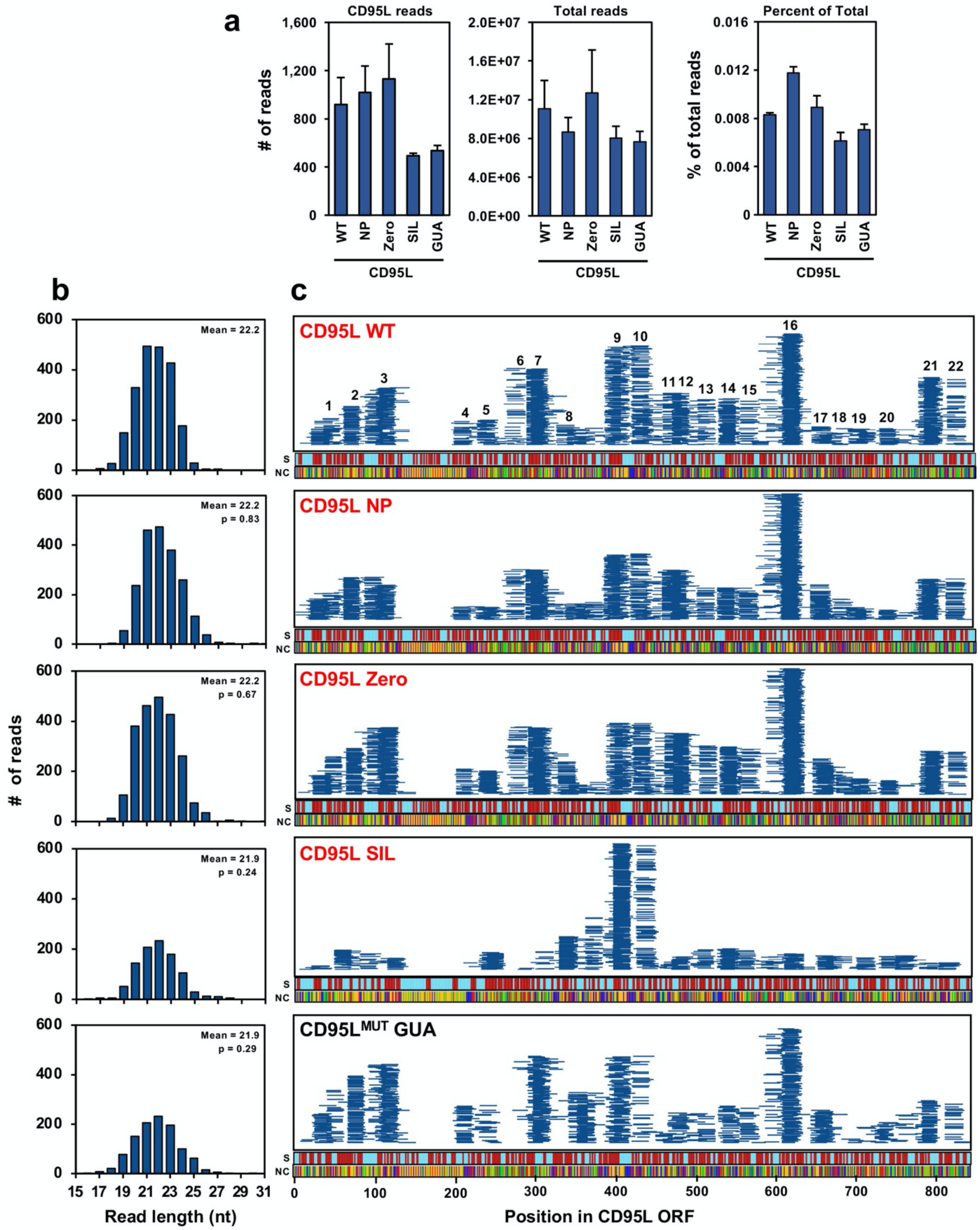

Figure S9 - Processing and RISC loading of CD95L mutant derived reads

RISC bound reads of CD95L mutants expressed in CD95 d.k.o. c12 cells were analyzed. **(a)** *Left*, raw counts of reads aligning to CD95L mutant sequences. *Center*, total raw reads sequenced per sample. *Right*, percentage of total raw reads that are derived from pLenti-CD95L mutants. The average of two replicates is shown. Error bars represent the SE of the mean. **(b)** Bar plots representing the read lengths of various CD95L derived reads in the RISC. Kruskal-Wallis p-values are given. **(c)** Mapping of CD95L-derived sRNAs along the ORF of each CD95L mutant. Each horizontal line represents one read. Reads from both replicates are displayed. Toxic mutants are labeled in red and non-toxic mutants in black. Beneath each stack plot, stem-loop regions (S) are mapped with stems (red) and loops (blue). The next bar below represents the nucleotide content of each CD95L mutant sequence, **A**denine (blue), **U**racil (green), **G**uanine (red), and **C**ytosine (yellow).
