# Supplementary figures and images for: CD95/Fas ligand mRNA is toxic to cells through more than one mechanism
Source: Mol Biomed. 2023 Apr 15;4:11. doi: 10.1186/s43556-023-00119-1 (PMC10105004; doi:10.1186/s43556-023-00119-1)

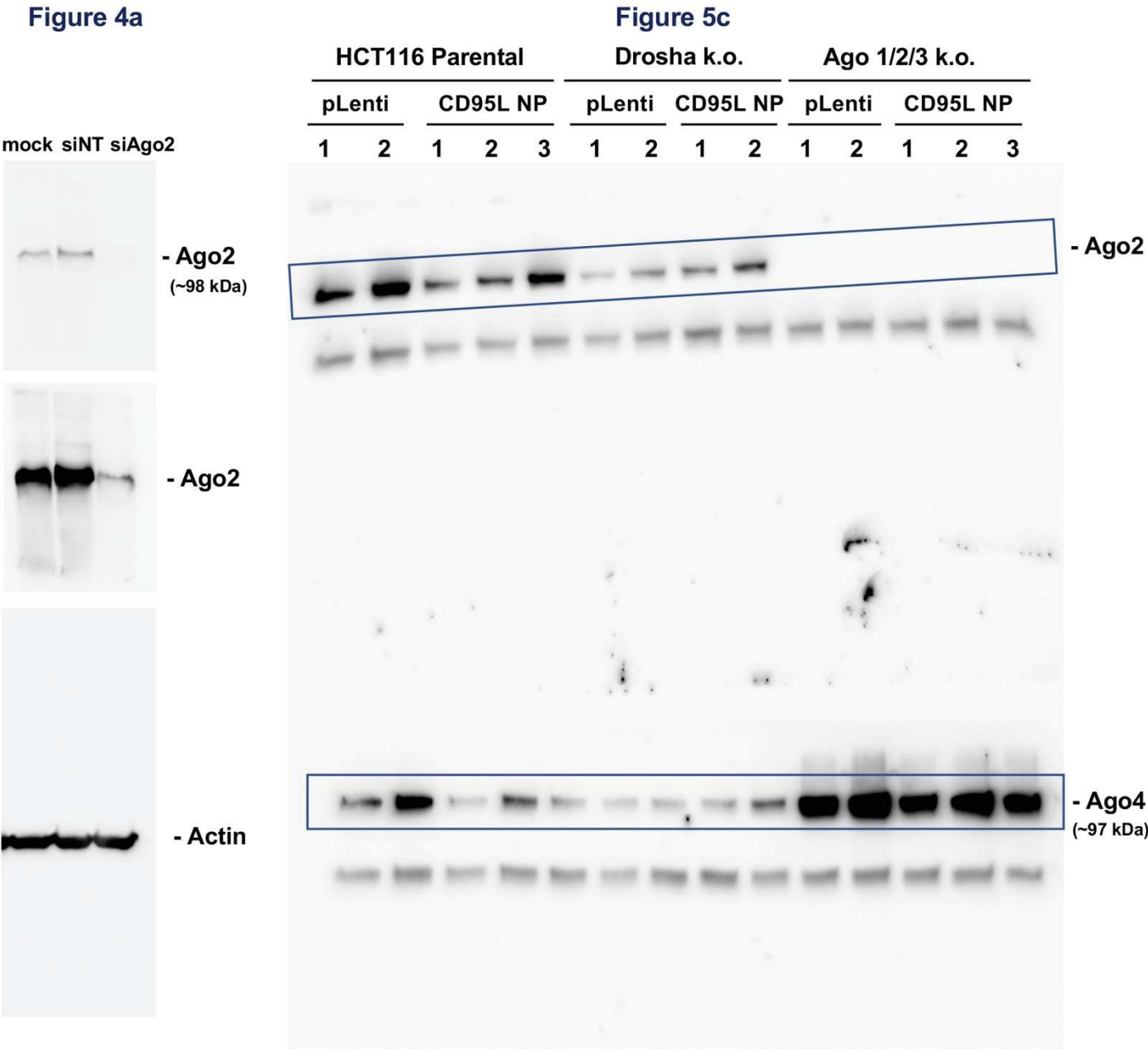

Figure S10 - Uncropped Western blots of Figure 4a and 5c

Supplement: Supplementary file 10 — Additional file 10: Supplementary Fig. 10. Uncropped Western blots of Figure 4a and 5c. [file 43556_2023_119_MOESM10_ESM.pdf]
